# A double-negative feedback loop between NtrBC and a small RNA rewires nitrogen metabolism in legume symbionts

**DOI:** 10.1128/mbio.02003-23

**Published:** 2023-10-18

**Authors:** Natalia I. García-Tomsig, Fernando M. García-Rodriguez, Sabina K. Guedes-García, Vicenta Millán, Anke Becker, Marta Robledo, José I. Jiménez-Zurdo

**Affiliations:** 1 Structure, Dynamics and Function of Rhizobacterial Genomes (RhizoRNA Lab), Estación Experimental del Zaidín, Consejo Superior de Investigaciones Científicas (CSIC), Granada, Spain; 2 Center for Synthetic Microbiology (SYNMIKRO), Philipps-Universität Marburg, Marburg, Germany; Yale School of Medicine, New Haven, Connecticut, USA; John Innes Centre, Norwich, United Kingdom

**Keywords:** riboregulation, rhizobia, alpha-proteobacteria, nitrogen fixation, *Sinorhizobium meliloti*, two-component regulatory systems

## Abstract

The nitrogen (N) status transduced *via* the NtrBC two-component system is a major signaling cue in the root nodule endosymbiosis of diazotrophic rhizobia with legumes. NtrBC is upregulated in the N-limiting rhizosphere environment at the onset of nodulation but silenced in nodules to favor the assimilation of the fixed N into plant biomass. We reported that the *trans*-acting sRNA NfeR1 (Nodule Formation Efficiency RNA) broadly influences the symbiotic performance of the α-rhizobium *Sinorhizobium meliloti*. Here, we show that NfeR1 is indeed an N-responsive sRNA that fine-tunes NtrBC output during the symbiotic transition. Biochemical and genetic approaches unveiled that NtrC and the LysR-type symbiotic regulator LsrB bind at distinct nearby sites in the NfeR1 promoter, acting antagonistically as repressor and activator of transcription, respectively. This complex transcriptional control specifies peak NfeR1 steady-state levels in N-starved and endosymbiotic bacteria. Furthermore, NfeR1 base pairs the translation initiation region of the histidine kinase coding mRNA *ntrB*, causing a decrease in both NtrB and NtrC abundance as assessed by double-plasmid genetic assays. In the context of endogenous regulation, NfeR1-mediated *ntrBC* silencing most likely amends the effective strength of the known operon autorepression exerted by NtrC. Accordingly, a lack of NfeR1 shifts the wild-type NtrBC output, restraining the fitness of free-living rhizobia under N stress and plant growth upon nodulation. The mixed NtrBC-NfeR1 double-negative feedback loop is thus an unprecedented adaptive network motif that helps α-rhizobia adjust N metabolism to the demands of an efficient symbiosis with legume plants.

**IMPORTANCE:**

Root nodule endosymbioses between diazotrophic rhizobia and legumes provide the largest input of combined N to the biosphere, thus representing an alternative to harmful chemical fertilizers for sustainable crop production. Rhizobia have evolved intricate strategies to coordinate N assimilation for their own benefit with N_2_ fixation to sustain plant growth. The rhizobial N status is transduced by the NtrBC two-component system, the seemingly ubiquitous form of N signal transduction in Proteobacteria. Here, we show that the regulatory sRNA NfeR1 (nodule formation efficiency RNA) of the alfalfa symbiont *Sinorhizobium meliloti* is transcribed from a complex promoter repressed by NtrC in a N-dependent manner and feedback silences *ntrBC* by complementary base-pairing. These findings unveil a more prominent role of NtrC as a transcriptional repressor than hitherto anticipated and a novel RNA-based mechanism for NtrBC regulation. The NtrBC-NfeR1 double-negative feedback loop accurately rewires symbiotic *S. meliloti* N metabolism and is likely conserved in α-rhizobia.

## INTRODUCTION

Combined nitrogen (N) is one of the most important limiting nutrients in agricultural soils. Under N starvation, certain α- and β-proteobacteria, generically called rhizobia, have the unique ability to elicit the morphogenesis of nodules upon infection of the roots (exceptionally the stems) of specific legume plants (1). Within the root nodules, invading rhizobia are accommodated intracellularly as morphologically differentiated bacteroids that support the nitrogenase-mediated reduction of the inert atmospheric dinitrogen (N_2_) to ammonia usable by the plant (2, 3). N availability is a major environmental cue signaling all developmental steps of these mutualistic rhizobia-legume symbioses in both bacteria and host plants (2, 4–7). As in most Proteobacteria, the master regulator of the rhizobial N stress response (NSR) is the classical NtrBC two-component system (TCS) (8, 9). Environmental N-limiting conditions promote phosphorylation of the transcription factor (TF) NtrC by the histidine kinase of the system, NtrB. In turn, NtrC-P binds RpoN (σ^54^)-dependent promoters to activate transcription of an array of genes specifying N assimilation through the glutamine synthetase (GS)-glutamate synthase (GOGAT) pathways (10, 11). Conversely, N surplus prevents NtrB autophosphorylation while activating its phosphatase activity to keep NtrC in a likely inactive dephosphorylated state. NtrBC-promoted N assimilation operates in soil and during early nodulation stages but is switched off in mature bacteroids to favor release of the fixed N to the host (2, 5, 12).

Repression of gene promoters by NtrC has been more rarely reported but actually occurs as a means of negative autoregulation of *ntrBC* in several bacteria, including rhizobia (10, 13–17). Such feedback regulation of bacterial gene control systems, either positive or negative, is a widespread mechanism that improves performance and guarantees robustness of environmental signal transduction (18, 19). Nonetheless, fine-tuning of the transcription output can be exerted by a second regulatory element, protein or small non-coding RNA (sRNA), arranged with the TF into a double-feedback loop, thereby involving mutual regulation of the regulators. Several mixed autoregulatory motifs consisting of a TF and a *trans*-acting sRNA have been already related to the control of bacterial outer membrane remodeling, metabolic adaptations to nutritional shifts, or the quorum sensing response (20–22). These logics rely on the TF-mediated transcriptional repression or activation of the sRNA, and post-transcriptional silencing of the TF mRNA by the sRNA. The latter is canonically exerted by a short antisense interaction at the translation initiation region assisted by RNA chaperones (e.g*.,* Hfq or ProQ) that abrogates translation and promotes decay of the TF mRNA (23).

In rhizobia, the vast majority of *trans*-sRNAs functionally characterized to date have been identified in the alfalfa (*Medicago sativa* L.) symbiont *Sinorhizobium meliloti* (24–26). One of those, referred to as nodule formation efficiency RNA (NfeR1), exists as an Hfq-independent 123-nt transcript widely conserved in phylogenetically related rhizobia, being the founding member of the so-called αr14 family of α-proteobacterial sRNAs (27, 28). The highest NfeR1 levels have been detected in bacteria growing exponentially in defined glutamate/mannitol medium (MM) and upon a salt shock. Glutamate is regarded as a poor N source and, therefore, MM formulated with this amino acid likely imposes N stress on bacteria (5, 10). Of note, NfeR1 is also markedly upregulated throughout the symbiotic interaction, that is, rhizoplane colonization, root hair infection, bacteroid differentiation, and N_2_ fixation. Consistently with this accumulation profile, *nfeR1* knockout compromises the osmoadaptation of free-living bacteria, nodulation kinetics, nodule development, and symbiotic efficiency of *S. meliloti* on alfalfa roots (28). A first reported alignment of the promoter regions of NfeR1 homologs encoded by diverse *Sinorhizobium*, *Rhizobium*, *Agrobacterium,* and *Brucella* representatives evidenced the recognizable α-proteobacterial -35/-10 RpoD (σ^70^) signature, CTTAGAC-N_17_-CTATAT, and a conserved upstream 29-nt stretch that is likely the major determinant of NfeR1 upregulation under stress and symbiotic conditions (28). However, the transcriptional regulation of NfeR1 has not been investigated in more detail, yet.

Members of the αr14 family of sRNAs share a predicted secondary structure consisting of three hairpins, each carrying the identical and ultraconserved “CCUCCUCCC” anti-Shine-Dalgarno (aSD) motif in their unpaired regions, while their respective stems differ highly in the primary nucleotide sequences (27, 29). Computational predictions with the IntaRNA and CopraRNA tools anticipated that all three motifs are equally competent to target multiple mRNAs by the canonical antisense mechanism referred to above (28). Most of the NfeR1 target mRNA candidates encode ABC transport systems, which is reminiscent of nutrient uptake regulation by the so-called AbcR sRNAs characterized in *S. meliloti* and several other α-proteobacteria (30, 31). However, NfeR1-mediated regulation of only two of those mRNAs, *SMc03121* and *SMb20442*, both encoding the periplasmic component of yet uncharacterized ABC transporters, has been experimentally tested and confirmed by a double-plasmid reporter assay. These experiments further revealed that the three NfeR1 aSD motifs do have a redundant function in target mRNA silencing (28).

Here, we show that NfeR1 is indeed an N stress-induced sRNA whose transcription is activated by the LysR-type symbiotic regulator LsrB and repressed by NtrC. Furthermore, NfeR1 silences the *ntrBC* mRNA by base-pairing at the *ntrB* translation initiation region *via* the aSD seeds, thereby counteracting NtrC-mediated autorepression. Disabling this double-negative feedback loop by *nfeR1* deletion downregulates NtrBC in free-living *S. meliloti* bacteria while upregulating the system in endosymbiotic bacteroids. These findings explain, at least partially, the *S. meliloti* symbiotic phenotype associated with NfeR1 loss-of-function.

## RESULTS

### The transcriptional regulators NtrC and LsrB bind the NfeR1 promoter

**Fig 1:**
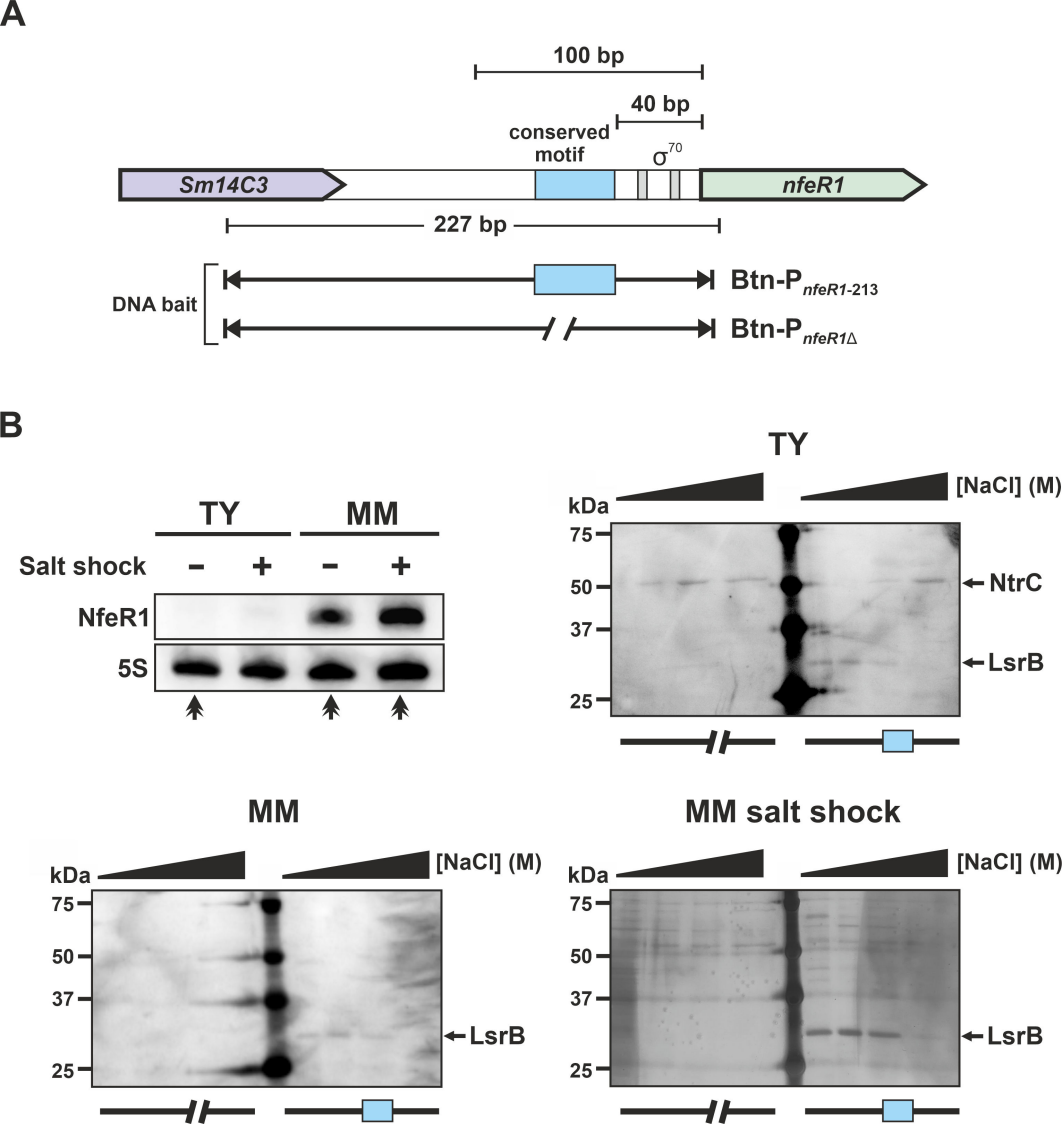
Affinity chromatography pull-down assay with P*_nfeR1_*. (**A**) Scheme of the NfeR1 promoter region containing the σ^70^ binding site (gray boxes) and the conserved motif responsible for P*_nfeR1_* induction (blue box). Biotinylated DNA fragments used as probes in the assays are depicted (Btn-P*_nfeR1_*_-213_ and Btn-P*_nfeR1_*_Δ_). The control DNA bait lacks the previously identified conserved motif. (**B**) Top left panel: Northern blot probing of NfeR1. Total RNA was obtained from Sm2B3001 cultured in the conditions indicated along the top of the panel. The 5S rRNA was probed as an RNA loading control. Lysates for pull-down assays were obtained from cultures in the conditions indicated by arrows, that is, TY, MM, and MM upon salt shock (400 mM for 1 h). DNA-bound proteins were eluted from columns with increasing concentrations of NaCl (0.3, 0.8, 1.5, and 2 M) and analyzed by SDS-PAGE followed by silver staining (upper right and bottom panels). Precision Plus Protein Dual Color Standard was run in the middle lane of the gels.

To identify putative regulatory proteins that bind the NfeR1 promoter region (P*_nfeR1_*), we first carried out affinity chromatography pull-down assays on streptavidin columns with biotinylated DNA fragments (Fig. 1A). The DNA bait was a 227 bp fragment (P*_nfeR1-213_*) extending from nucleotide positions −213 to +14 relative to the NfeR1 transcription start site (TSS). As a control we used P*_nfeR1_*_Δ_, which contains a 60-nt deletion corresponding to the conserved motif unveiled by the reported promoter alignment (positions −40 to −100 in P*_nfeR1-213_*) (28). Biotinylated P*_nfeR1-213_* and P*_nfeR1_*_Δ_ probes were mixed with lysates from strain Sm2B3001 grown exponentially in a complete TY medium, MM-defined medium, or upon a salt shock imposed in MM (Fig. 1B). The latter two conditions promote endogenous upregulation of NfeR1, whereas the transcript is hardly detected in TY cultures, as confirmed by repeating the Northern hybridization experiment of our previous study (Fig. 1B; upper-left panel) (28). Proteins bound to both promoter fragments were eluted from columns by the addition of increasing concentrations of NaCl and further analyzed qualitatively by SDS-PAGE (Fig. 1B; upper-right and lower panels) and liquid chromatography-mass spectrometry (LC-MS/MS). This analysis reliably identified the LysR-type transcriptional regulator (LTTR) LsrB (36 kDa) as the protein bound to P*_nfeR1-213_* but not to the control P*_nfeR1_*_Δ_ in all culture conditions. In addition, the TF NtrC (54 kDa) was identified as a binding partner of both P*_nfeR1-213_* and P*_nfeR1_*_Δ_, specifically in bacteria grown in TY, where NfeR1 is markedly downregulated. These findings suggest the binding of NtrC either upstream or downstream of the deleted promoter motif, which is likely recognized by LsrB.

Therefore, we performed a new alignment with sequences extending up to 213-nt upstream of the TSS of several NfeR1 homologs in *Sinorhizobium* and *Rhizobium* species (Fig. S1). A more detailed inspection of the −72 to −60-nt stretch unveiled that the conserved motif TGCA-N_6_-GCAT meets the generic LTTR T-N_11_-A box, closely matching the LsrB binding site identified in the promoter of the AbcR1 sRNA (31). This alignment also evidenced the putative NtrC binding motif TGC-N_11_-GCA between positions −101 and −116 in *S. meliloti* P*_nfeR1_*, which has been described in both NtrC activated and repressed promoters (14, 32, 33). Besides, the conserved A/T-rich region within this plausible NtrC binding site has been also reported in gene promoters regulated by NtrC in *Salmonella* sp (32). The NtrC signature was not evident when the alignment was extended with NfeR1 promoter sequences from α-proteobacterial plant or mammal pathogens, for example*, Agrobacterium tumefaciens* or *Brucella* sp. (not shown), which suggests that conservation of this promoter architecture is restricted to plant symbionts.

**Fig 2:**
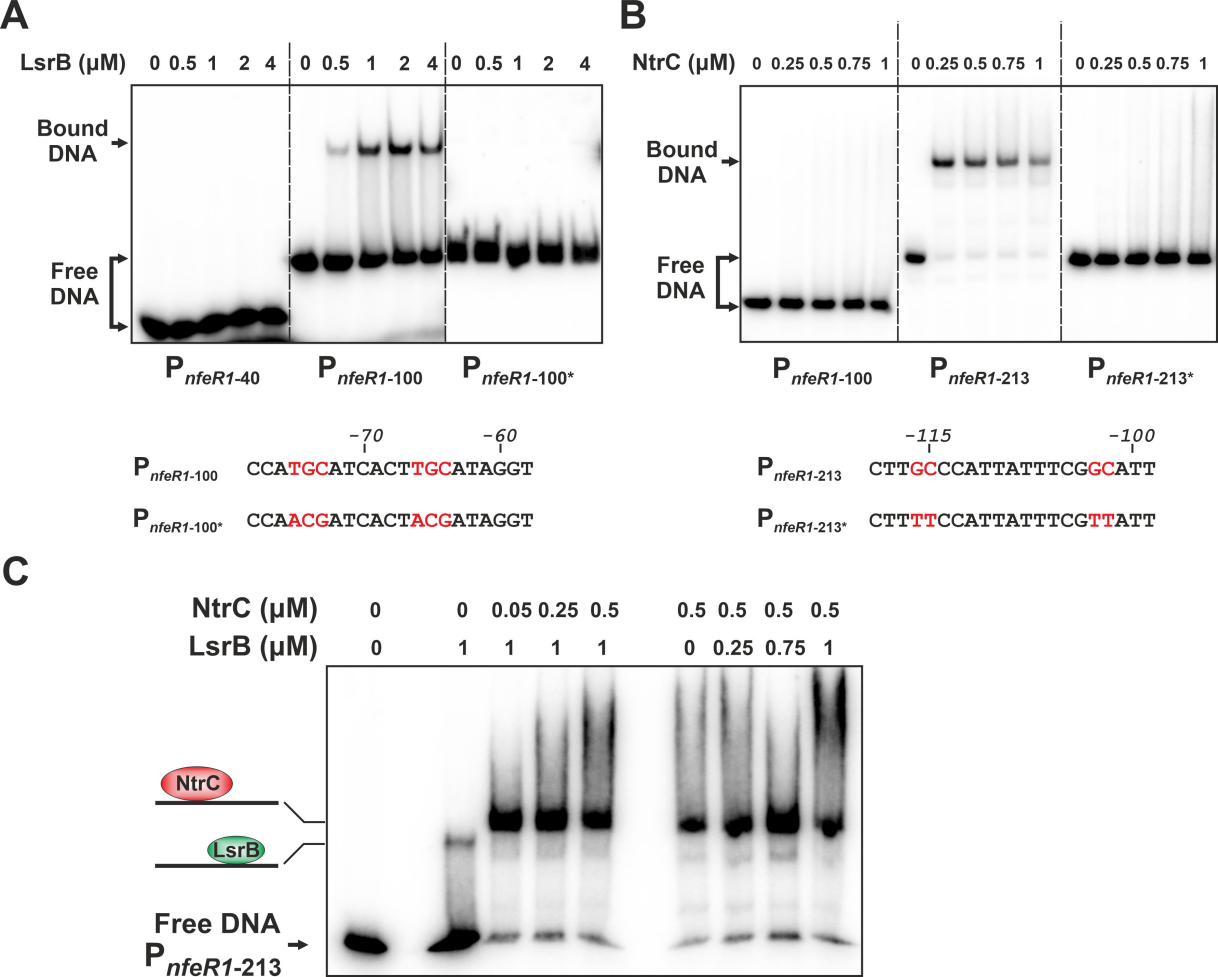
LsrB and NtrC binding to the NfeR1 promoter *in vitro*. (**A**) Gel shift assays with radiolabeled P*_nfeR1-40_* (40 bp), P*_nfeR1-100_* (100 bp), and P*_nfeR1-100_* mutant variant (P_nfeR1-100*_) incubated with increasing concentrations of purified LsrB as indicated on top of the panel. Nucleotide substitutions for generation of P_nfeR1-100*_ are marked in red in the sequences below the panel. Numbers indicate nucleotide positions relative to the NfeR1 TSS. (**B**) Gel shift assays with radiolabeled P*_nfeR1-100_* (100 bp), P*_nfeR1-213_* (213 bp), and its mutant variant (P_nfeR1-213*_) incubated with increasing concentrations of purified NtrC as indicated on top. Point mutations to generate P_nfeR1-213*_ are marked in red in the sequences below. Nucleotide positions relative to the NfeR1 TSS are also indicated. (**C**) NtrC outcompetes LsrB for binding to P*_nfeR1_*. Gel shifts assay with radiolabeled P*_nfeR1-213_* incubated with purified LsrB (1 µM) or NtrC (0.5 µM) and increasing concentrations of the other protein as indicated on top of the panel. Diagrams of the detected protein-DNA complexes are depicted to the left.

To further assess LsrB and NtrC binding to P*_nfeR1_*, we performed an electrophoretic mobility shift assay (EMSA) with different radiolabeled DNA fragments of the promoter region (Fig. 2). These experiments unambiguously revealed LsrB binding to a 100-nt long fragment (P*_nfeR1_*_-100_) (Fig. 2A). However, both trimming of (P*_nfeR1-_*_40_) and point mutations in (P*_nfeR1_*_-100*_), the proposed LsrB binding motif prevented the formation of DNA-protein complexes. On the other hand, incubation with NtrC resulted in an electrophoretic mobility shift of P*_nfeR1_*_-213_ but not of P*_nfeR1_*_-100_, consistently with the predicted location of the NtrC interaction site (Fig. 2B). Indeed, nucleotide substitutions in this motif also abrogated binding of the regulator to P*_nfeR1_*_-213_. DNA footprinting further uncovered a nucleotide stretch protected against DNase I digestion that maps the NtrC binding site between positions −95 and −121 at P*_nfeR1_*, as expected (Fig. S2). Interestingly, co-incubation of P*_nfeR1_*_-213_ with both recombinant proteins at different combinations and concentrations evidenced that NtrC always outcompeted LsrB for binding (Fig. 2C). Collectively, these data anticipate that NtrC and LsrB regulate NfeR1 transcription by binding to nearby promoter sites with very different affinity.

### LsrB activates, whereas NtrC represses NfeR1 transcription

**Fig 3:**
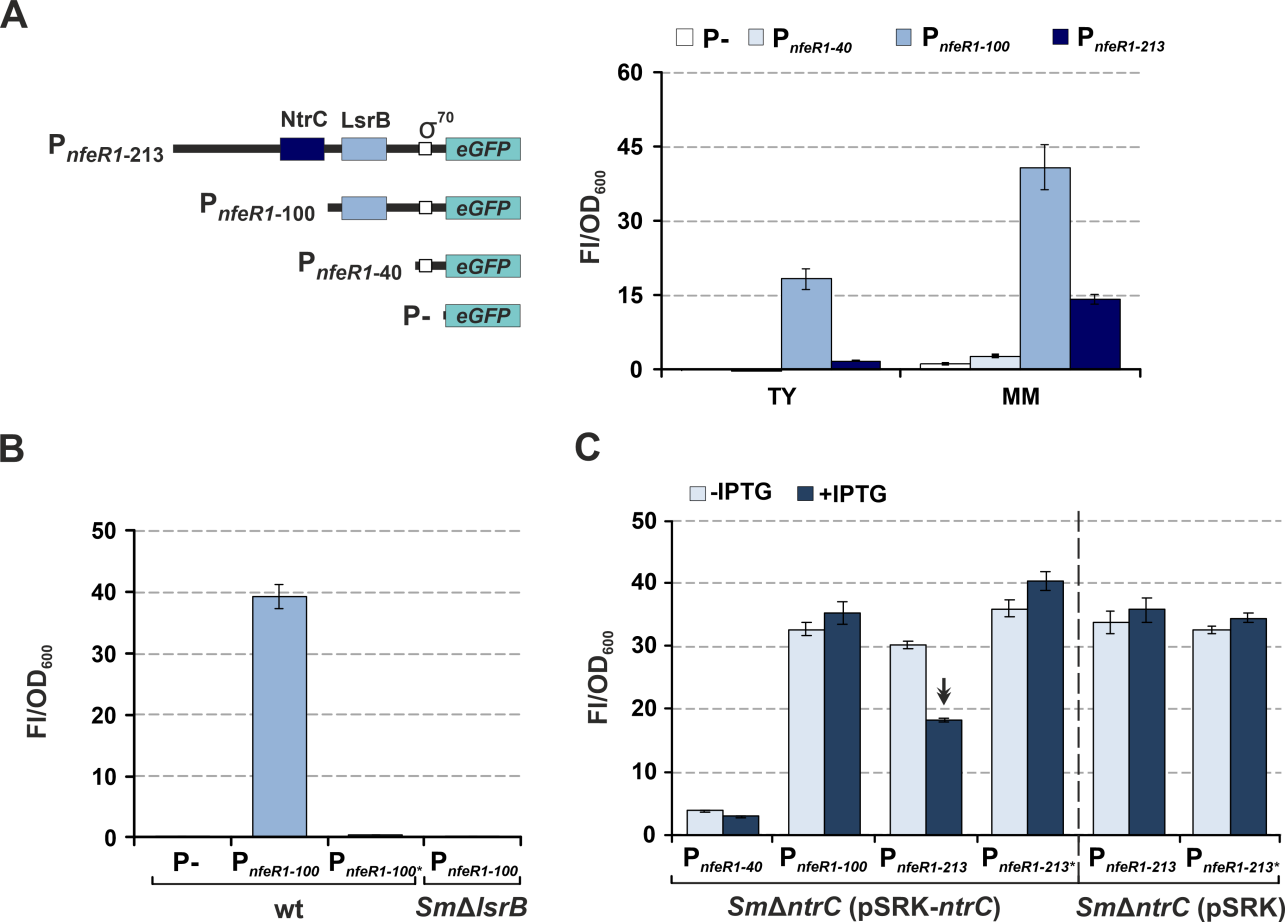
Transcriptional regulation of NfeR1 by LsrB and NtrC assessed by promoter-*eGFP* fusions. (**A**) Fluorescence derived from full-length and trimmed versions of P*_nfeR1_*, as diagrammed, was determined in Sm2B3001 growing in TY or MM. P-, no promoter motifs. (**B**) LsrB-dependent P*_nfeR1_* activity. P*_nfeR1-100_*-derived fluorescence was measured in wild-type and LsrB mutant (SmΔ*lsrB*) strains. P_nfeR1-100*_ contains the point mutations described in Fig. 2. (**C**) NtrC-dependent P*_nfeR1_* activity. Fluorescence derived from full-length (P*_nfeR1-_*_213_), trimmed (P*_nfeR1-_*_100_, P*_nfeR1-_*_40_), and mutant (P*_nfeR1-_*_213*_) versions of P*_nfeR1_* was determined in SmΔ*ntrC* transformed with pSRK-*ntrC* or the empty vector (pSRK) as control. Reporter bacteria were cultured in MM with or without isopropyl-β-D-thiogalactopyranoside (IPTG). The double arrowhead highlights the repression of the promoter by NtrC. Values reported in all plots are means and SD of nine fluorescence measurements normalized to the OD_600_ of cultures (Fl/OD_600_), that is, three replicates of three independent cultures of each reporter strain.

We therefore investigated genetically the effects of LsrB and NtrC on NfeR1 transcription *in vivo*. For that, we fused DNA fragments encompassing 213, 100, and 40-nt upstream the NfeR1 TSS to a promoterless *eGFP* (P-*eGFP*), namely P*_nfeR1_*_-213_, P*_nfeR1_*_-100_, and P*_nfeR1_*_-40_*,* respectively, in the single-copy plasmid pABCa (Fig. 3A). The latter two fragments were generated by sequential trimming of the probable NtrC and LsrB binding sites in P*_nfeR1_*_-213_. These reporter transcriptional fusions were independently mobilized to Sm2B3001, and fluorescence was scored in TY and MM cultures. As expected, the transconjugants carrying the construct lacking both the NtrC and LsrB binding motifs (P*_nfeR1_*_-40_*::eGFP*) yielded hardly detectable fluorescence, whereas the highest *eGFP* expression was evident in bacteria harboring P*_nfeR1_*_-100_*::eGFP* in both culture conditions. Although the endogenous NfeR1 transcript is barely detected in bacteria grown in TY medium (Fig. 1B; Northern blot), ectopic *eGFP* transcription from P*_nfeR1_*_-100_ (devoid of the NtrC signature) was far above that of P*_nfeR1_*_-40_ in this condition, indicating active LsrB-dependent NfeR1 transcription. P*_nfeR1_*_-213_-derived fluorescence decreased by ~10-fold with respect to that from P*_nfeR1_*_-100_ in TY broth, which evidences a marked transcriptional repression of the promoter by NtrC in this culture condition. In MM, this relative repression was scarcely ~2.4-fold, rendering activity of the full-length promoter (P*_nfeR1_*_-230_) eightfold higher in bacteria grown in defined than in complete TY medium, which supports the abundance profile of NfeR1 revealed by Northern hybridization.

We next transformed the Sm2B3001 derivatives lacking LsrB and NtrC (SmΔ*lsrB* and SmΔ*ntrC*) with the reporters of wild-type P*_nfeR1_* and its mutant variants, that is, P*_nfeR1-_*_100_/P*_nfeR1_*_-100*_ or P*_nfeR1_*_-213_/P*_nfeR1_*_-213*_ (Fig. 3B and C). Both deletion of *lsrB* and nucleotide substitutions at the LsrB binding site (P*_nfeR1_*_-100*_) abrogated transcription from P*_nfeR1_*_-100_, as inferred from the fluorescence of log MM cultures of the reporter strains (Fig. 3B). Deletion of *ntrC* precludes *S. meliloti* growth in MM. Thus, to further assess NtrC-dependent repression of NfeR1 transcription, SmΔ*ntrC* reporter strains were also complemented with pSRK-*ntrC*, which contains *lacI* and allows *ntrC* expression from an IPTG inducible *lac* promoter (34). Acceptable yet suboptimal growth of these bacteria in MM in the absence of the inducer is likely sustained by leaky *ntrC* transcription. Fluorescence of this set of SmΔ*ntrC* double transconjugants was then measured in log MM cultures (Fig. 3C). The addition of IPTG to the cultures, that is*,* the presence of NtrC, resulted in a significant decrease in P*_nfeR1_*_-213_-derived fluorescence, while neither trimming of (P*_nfeR1_*_-100_) nor point mutations in (P*_nfeR1_*_-213*_) the NtrC binding motif altered fluorescence values. Similarly, the fluorescence of SmΔ*ntrC* strains harboring *eGFP* fusions to P*_nfeR1_*_-213_ or P*_nfeR1_*_-213*_, and co-transformed with the empty pSRK vector was not influenced by IPTG induction. Together, these data indicate that LsrB is indispensable for NfeR1 expression in free-living *S. meliloti* bacteria, whereas NtrC acts as a transcriptional repressor. These regulatory activities are most likely exerted by binding of both TFs *in vivo* to the respective proposed promoter motifs.

On the other hand, NfeR1 is highly abundant in nodule tissues, which is supported by strong transcription rates during the symbiotic transition, as already tracked by promoter-reporter fusions (28). However, in those assays, we used a short version of the promoter (P*_nfeR1_*_-100_) devoid of the, at that time unknown, NtrC-binding site. Therefore, we re-evaluated NfeR1 transcription *in planta* using the P*_nfeR1-213_::eGFP* fusion in the wild-type, SmΔ*lsrB,* and SmΔ*ntrC* genetic backgrounds (Fig. S3). Confocal microscopy confirmed that the full-length P*_nfeR1_* is also highly active in the rooting solution (Fig. S3A), infection threads (Fig. S3B), and N_2_-fixing bacteroids isolated from mature nodules (Fig. S3C) elicited by wild-type bacteria. As expected, the lack of LsrB strongly hampered, whereas NtrC did not apparently influence transcription from P*_nfeR1_* at the onset of nodulation and within nodules. These findings confirm that the complex regulation at P*_nfeR1_* does guarantee high NfeR1 levels throughout the symbiotic interaction of *S. meliloti* with alfalfa.

### N stress promotes upregulation of NfeR1

**Fig 4:**
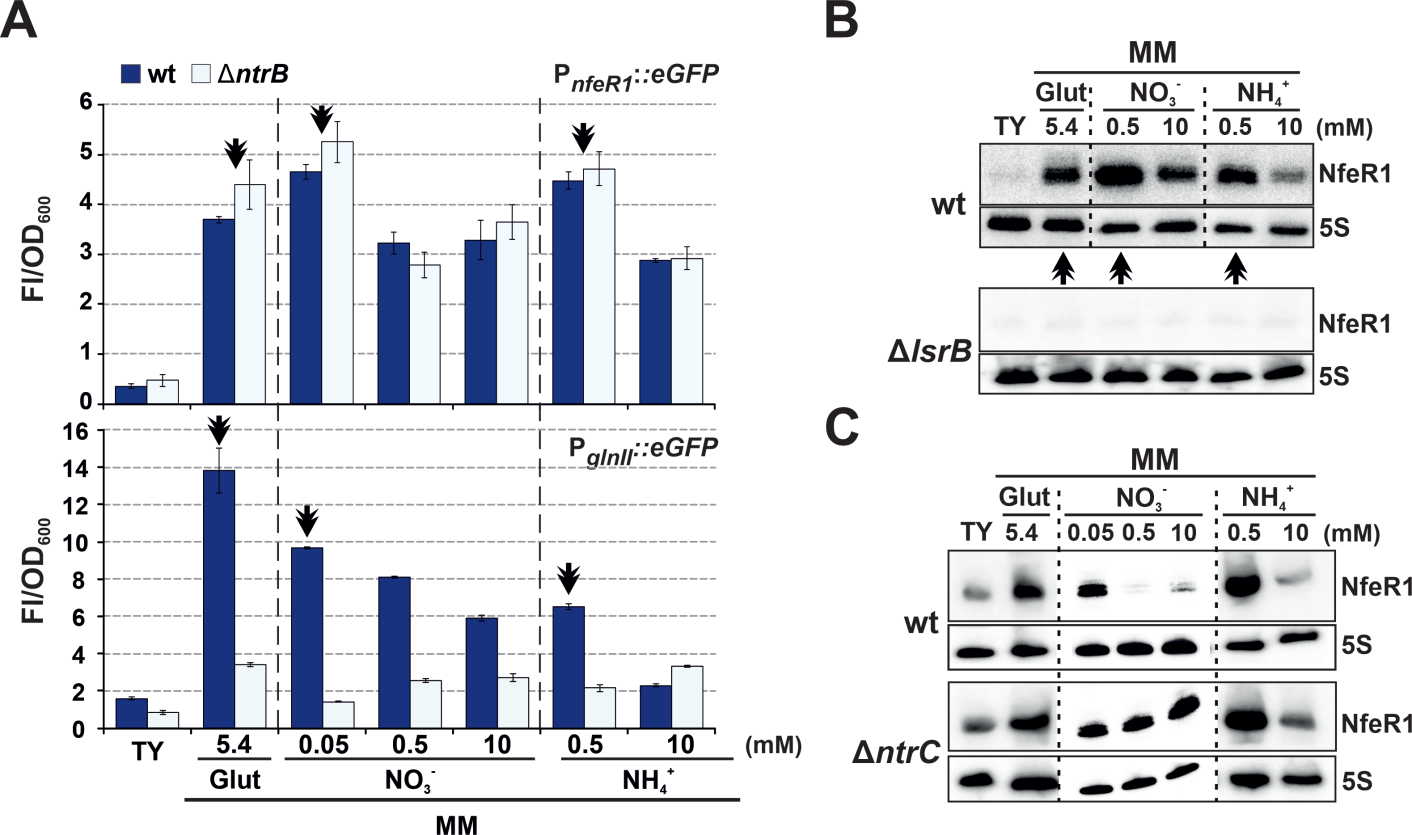
NfeR1 is a N stress-induced sRNA. (**A**) The activity of NfeR1 and *glnII* promoters under N stress. Fluorescence derived from P*_nfeR1_::eGFP* and P*_glnII_::eGFP* fusions was determined in Sm2B3001 (wild type) and SmΔ*ntrB* growing in MM supplemented with different N sources and at the indicated concentrations. TY is regarded as a rich medium (N surplus). Double arrowheads indicate conditions of maximum activity of both promoters. Plotted values are means and SD of nine fluorescence measurements normalized to the OD_600_ of cultures (Fl/OD_600_), that is, three replicates of three independent cultures of each reporter strain. (**B**) Northern blot analysis of N-dependent NfeR1 expression. Total RNA was obtained from Sm2B3001 or SmΔ*lsrB* cultured in conditions indicated along the top of the panel. Double arrowheads indicate conditions that promote the highest NfeR1 accumulation. (**C**) Northern blot analysis of NfeR1 regulation by NtrC. Prior to total RNA extraction, Sm2B3001 or SmΔ*ntrC* was grown in TY to exponential phase, washed in PBS, and cultured in the indicated conditions for 4 h. The 5S rRNA was probed as an RNA loading control.

Since the NtrBC TCS is the master regulator of the NSR, we reasoned that NfeR1 might respond to the *S. meliloti* N status. To test this hypothesis, strain Sm2B3001 and its *ntrB* deletion mutant (SmΔ*ntrB*) were transformed with the plasmid expressing the *eGFP* reporter fused to P*_nfeR1_*_-213_ (from here on P*_nfeR1_::eGFP*), and the transconjugants were grown in TY and MM, the latter formulated with glutamate, nitrate (MM-NO_3_), or ammonia (MM-NH_4_) at likely limiting or excess concentrations (Fig. 4A). A gene known to be activated by NtrC upon its phosphorylation by NtrB is *glnII*, which encodes the major *S. meliloti* glutamine synthetase GSII (7). Thus, as N stress reporter in these assays, we used the transcriptional fusion of the *glnII* promoter to *eGFP* (P*_glnII_::eGFP*). P*_glnII_* activity in SmΔ*ntrB* was thus considered the background of the NSR activation in each culture condition. Fluorescence from P*_nfeR1_::eGFP* was more than sevenfold higher in wild-type bacteria grown in the classical MM ( 6.5 mM glutamate) than in complete TY and decreased as the N concentration increased in MM-NO_3_ and MM-NH_4_. Remarkably, the highest transcription levels from P*_nfeR1_* were measured in conditions of N stress as reported by P*_glnII_*. Unlike that of P*_glnII_*, P*_nfeR1_* activity was not influenced by the *ntrB* knockout, suggesting that repression of NfeR1 transcription in TY and MM (Fig. 3A) does not require NtrB-dependent phosphorylation of NtrC and might depend on the oligomerization states of both LsrB and NtrC regulators in the different growth media.

Northern blot probing of RNA extracts from Sm2B3001 grown in the same conditions evidenced a correlation between NfeR1 accumulation and P*_nfeR1_* activity profiles, further confirming upregulation of NfeR1 under N stress (Fig. 4B). Besides, NfeR1 was undetectable in the SmΔ*lsrB* strain, indicating again that LsrB is indispensable for the expression of the sRNA in all the conditions tested. As stated above, lack of NtrC compromises *S. meliloti* growth under N stress. Thus, to assess the impact of NtrC on NfeR1 steady-state levels, Sm2B3001 and SmΔ*ntrC* bacteria were initially cultured in TY until exponential phase (OD_600_ 0.8), then washed in PBS solution, resuspended in different modified MM and grown for further 4 h in the fresh media (Fig. 4C). Probing of total bacterial RNA upon these treatments confirmed NfeR1 upregulation in the wild-type strain under N stress. In SmΔ*ntrC*, NfeR1 abundance was comparatively more similar among the tested conditions, with the transcript readily detected also under N surplus. We, therefore, conclude that LsrB activates and NtrC represses transcription from P*_nfeR1_* to render NfeR1 upregulated under N starvation.

### NfeR1 downregulates *ntrBC* by a canonical RNA silencing mechanism

**Fig 5:**
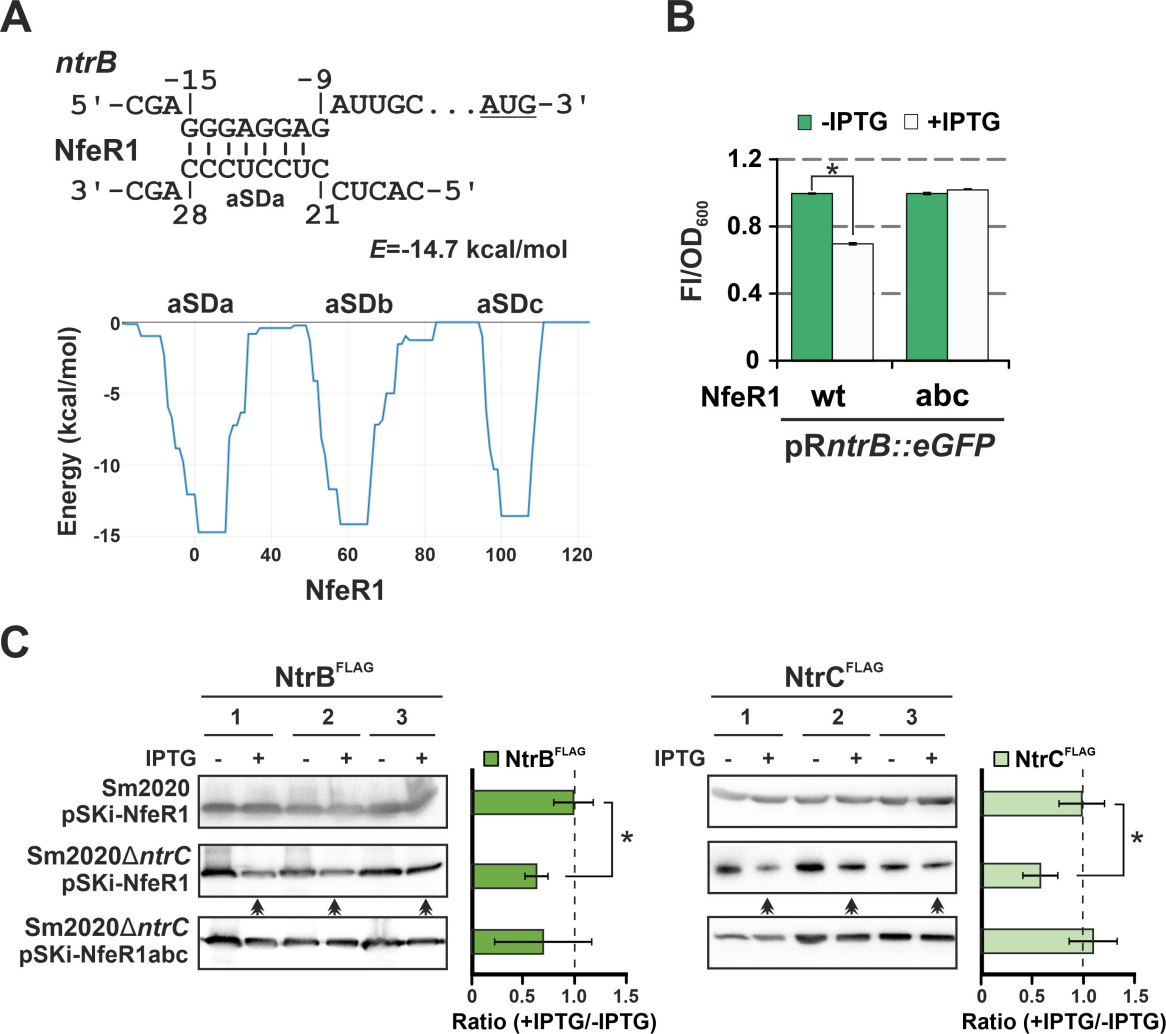
Post-transcriptional silencing of *ntrBC* by NfeR1. (**A**) IntaRNA predicted base-pairing interactions between NfeR1 aSDa-c and the *ntrB* mRNA (upper graph). The start codon of *ntrB* is underlined. Numbers denote nucleotide positions relative to the start codon of the *ntrB* mRNA and the NfeR1 TSS. The predicted minimum hybridization energy (*E*) between NfeR1-aSDa and the mRNA is indicated. Energy values for interactions involving aSDb and aSDc are similar (lower graph). (**B**) NfeR1 downregulates *ntrB*. Fluorescence of reporter strains co-transformed with the *ntrB::eGFP* translational fusion and plasmids overexpressing the wild-type NfeR1 or its mutant variant NfeR1abc upon IPTG-induction. Values plotted in the histogram correspond to the means and SD of 27 fluorescence measurements normalized to the OD_600_ of the cultures (Fl/OD_600_), that is, three determinations of three independent cultures from three independent double transconjugants for each reporter strain. (**C**) Silencing of *ntrB* influences NtrBC abundance. Western blot analysis of NtrB^FLAG^ and NtrC^FLAG^ produced constitutively from P*_syn_-ntrB^FLAG^* and P*_syn_-ntrBC^FLAG^* in Sm2020 or Sm2020Δ*ntrC* upon IPTG-induced expression of NfeR1 or its mutant variant NfeR1abc as indicated to the left. All gel lanes were loaded with equal protein amounts (OD_600_ equivalent to 0.05). Accumulation of the tagged proteins was evaluated 16 h after IPTG addition by quantification of band intensities corresponding to three independent cultures of three independent double transconjugants (1–3), and the ±IPTG ratios were plotted (histograms to the right). Double arrows indicate reduced NtrB^FLAG^ and NtrC^FLAG^ levels upon IPTG addition to cultures. Asterisks in the plots indicate significant differences according to the ANOVA test, *P* < 0.05.

Computational predictions anticipate a large NfeR1 regulon (28). Interestingly, among the multiple NfeR1 target candidates, we found the *ntrB* mRNA as directly related to the NSR regulation. The IntaRNA algorithm predicts similarly favored antisense interactions involving eight nucleotides within the translation initiation region of *ntrB* and any of the three functionally redundant NfeR1 aSD motifs, referred to as aSDa, aSDb, and aSDc (Fig. 5A). Of note, this interaction likely occurs also between NfeR1 homologs and *ntrB* in other α-rhizobia as predicted by CopraRNA (not shown). Therefore, we first assessed the impact of NfeR1 on NtrB translation by a double-plasmid reporter assay. For that, we constructed pR*ntrB::eGFP*, expressing a translational fusion of the *ntrB* 5′ region to *eGFP* from the synthetic constitutive promoter P*_syn_*, and its compatible plasmids pSKi-NfeR1 and pSKi-NfeR1abc. In these two latter constructs, the wild-type NfeR1 transcript or its NfeR1abc variant was placed under the control of a SinRI-based IPTG-inducible promoter (28, 35). NfeR1abc is mutated at the three aSD seeds, which fully disrupts the predicted base pairing with *ntrB*. Plasmid pR*ntrB::eGFP* was jointly mobilized with either pSKi-NfeR1 or pSKi-NfeR1abc to *S. meliloti* strain Sm2020, which carries deletions of the chromosomal *loci abcR1/2* and *sinRI*, to avoid possible interference in the assays of the NfeR1-related AbcR1/2 sRNAs and the endogenous quorum-sensing regulation (31, 35). Fluorescence of log TY cultures of the reporter strains significantly decreased upon IPTG-induced expression of NfeR1 but not of NfeR1abc (Fig. 5B), thus suggesting NfeR1-mediated downregulation of *ntrB* by primary blocking of translation upon base-pairing.

*ntrB* is likely co-transcribed with its flanking genes *dusB* (encoding the seemingly NSR-unrelated tRNA-dihydrouridine synthase B) and *ntrC* (Fig. S4A). However, previous differential RNAseq experiments unveiled a TSS preceding the NtrC coding sequence but, strikingly, failed to detect a TSS for the polycistronic mRNA (36). We therefore revised the expression outcome from this *S. meliloti* genomic region at the levels of transcription and protein production. First, we used fusions of putative *dusB* and *ntrC* promoters (P*_dusB_* and P_ntrC*_) to *eGFP* to measure their transcriptional activity in strain Sm2B3001 (Fig. S4A; left panel). Transcription from P*_dusB_* was robust in TY broth and increased in N-starving media (MM and 0.5 mM MM-NH_4_), whereas P_ntrC*_-derived fluorescence was barely detectable in any of the culture conditions. We next constructed plasmids to ectopically produce in Sm2B3001 a FLAG-tagged NtrC encoded either by the whole operon or a single gene transcribed from P*_dusB_* or P_ntrC*_, respectively (Fig. S4A; right panel). Western blot probing of protein extracts with anti-FLAG antibodies reliably detected NtrC^FLAG^ when expressed from P*_dusB_* but not from P_ntrC*_, also revealing an N-dependent accumulation profile of the protein correlating with the transcriptional activity of the promoter. Interestingly, EMSAs further confirmed the binding of NtrC to P*_dusB_* (Fig. S4B).

Together, these are additional experimental evidence to previously published data supporting transcription of *dusBntrBC* as a polycistronic mRNA and negative autoregulation of the operon by NtrC binding at P*_dusB_* (10, 15, 17). Therefore, blocking of NtrB translation upon NfeR1 base-pairing to *ntrB* might promote concomitant decay of the *ntrBC* mRNA, thereby influencing NtrC abundance. To test this hypothesis, plasmids expressing *ntrB^FLAG^* or *ntrBC^FLAG^* constitutively from P*_syn_* were mobilized to Sm2020 and its *ntrC* deletion mutant derivative (Sm2020Δ*ntrC*) previously transformed with pSKi-NfeR1 or pSKi-NfeR1abc (Fig. 5C). In these assays, transcription of *ntrBC* and NfeR1 was thus uncoupled from the endogenous regulation. Western blot analysis of the double transconjugants upon IPTG induction of NfeR1 and NfeR1abc expression in log TY cultures revealed downregulation of both NtrB^FLAG^ and NtrC^FLAG^ by the wild-type sRNA but not by its mutant variant (Fig. 5C). The decrease in protein abundance was modest but statistically significant, as expected from a *trans*-acting sRNA that adjusts gene expression post-transcriptionally. Strikingly, this effect was only evident in the Δ*ntrC* background, indicating interference of the chromosomally encoded NtrC on NfeR1-mediated regulation of the NtrBC TCS. Since plasmids expressing *ntrB^FLAG^* and *ntrC^FLAG^* are devoid of P*_dusB_*, this interference likely occurs by an unknown mechanism probably unrelated to NtrC-mediated transcriptional autorepression of the operon. Post-transcriptional silencing of *ntrBC* by NfeR1 thus closes a mixed (protein-sRNA) double-negative feedback loop for the regulation of this TCS in *S. meliloti*.

### Opposite effect of *nfeR1* deletion on *ntrBC* levels in free-living and nodule bacteria

**Fig 6:**
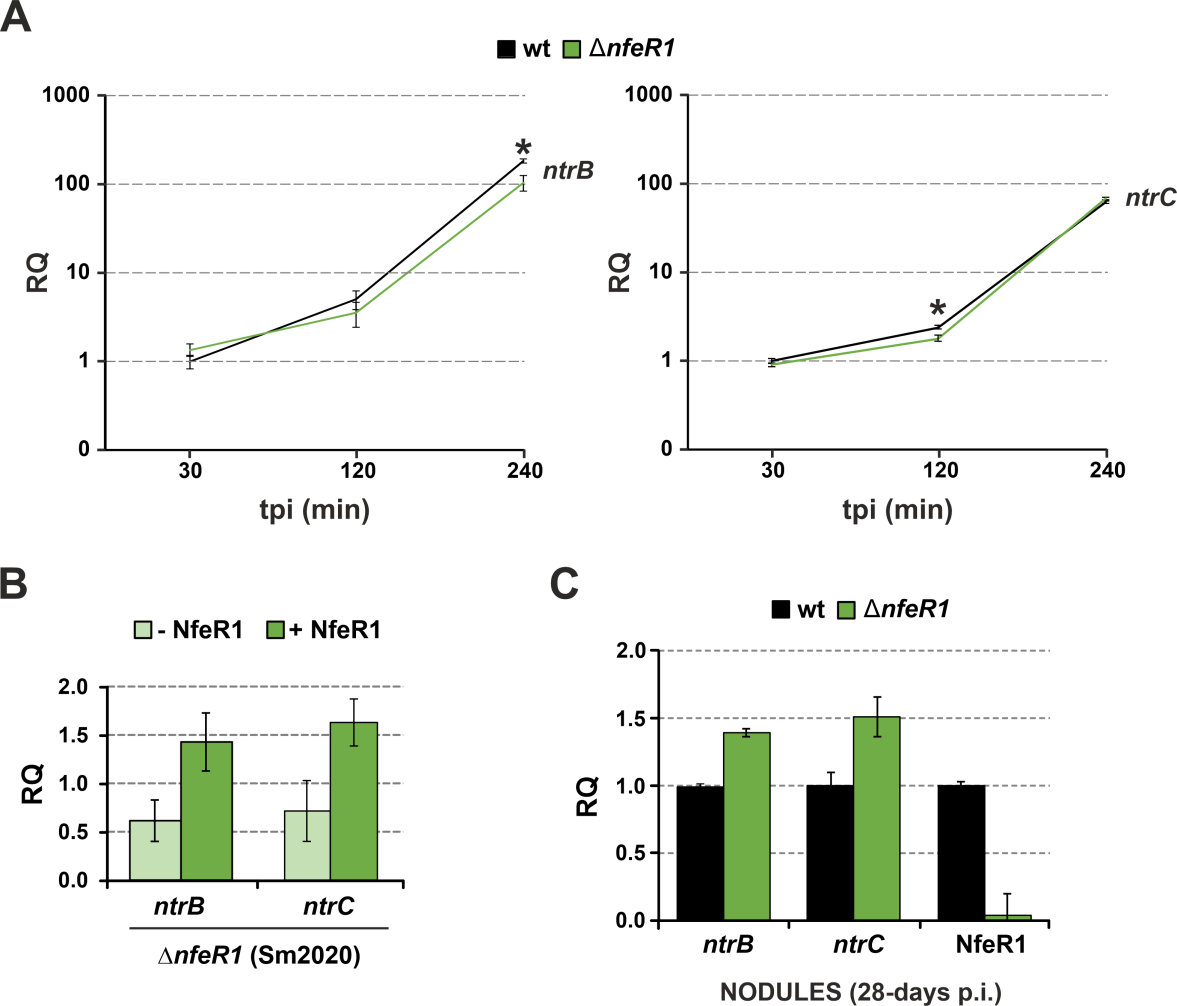
RT-qPCR analysis of NfeR1 impact in *ntrBC* levels. (**A**) NfeR1 is required for full expression of *ntrBC* in N-stressed free-living bacteria. RNA fractions were extracted from Sm2B3001 and SmΔ*nfeR1* grown in TY to exponential phase, washed in PBS, and cultured in MM to impose N stress for 30 min, 2 h, and 4 h (time post-induction; tpi). Values plotted are means and SD of two determinations in two independent cultures. Asterisks indicate significant differences according to the ANOVA test, *P* < 0.05. (**B**) Ectopic NfeR1-(over)expression promotes *ntrBC* accumulation. Total RNA was extracted from Sm2020 bacteria transformed with pSKiNfeR1 and cultured in MM to exponential phase. Three independent replicates were separated in IPTG-treated and untreated sub-cultures and cultured further for 1 h. Values plotted in the bar graphs correspond to the means and SD of all those determinations. (**C**) NfeR1 contributes to *ntrBC* silencing in bacteroids. RNA from mature wild-type or NfeR1 mutant nodules, that is*,* 28 days post-inoculation (p.i.), were analyzed by RT-qPCR to determine the accumulation levels of *ntrB* and *ntrC*. As a control, NfeR1 was only detected in RNA from wild-type nodules. In all experiments, relative quantification (RQ) values were normalized to *SMc01852* as a constitutive control.

We next investigated the impact of NfeR1 in the output of the NtrBC TCS in the endogenous regulatory background of both cultured and endosymbiotic *S. meliloti* bacteria. For that, we assessed the abundance of *ntrBC* in wild-type and mutant strains by RT-qPCR, using primer pairs specific for each cistron (Fig. 6). First, strain Sm2B3001 and its *nfeR1* deletion mutant SmΔ*nfeR1* were precultured in TY broth to log phase, and pelleted cells were subsequently suspended in MM to induce *ntrBC* expression by N stress (Fig. 6A). RT-qPCR on RNA extracts from cells withdrawn 30, 120, and 240 min upon medium shifting revealed the progressive accumulation of *ntrB* and *ntrC* in wild-type bacteria as expected. Interestingly, *nfeR1* knockout caused significant downregulation of both genes at certain time points, that is, 240 and 120 min for *ntrB* and *ntrC*, respectively. Of note, this effect was reversed by IPTG-induced expression of NfeR1 in log MM cultures of Sm2020 (i.e., *nfeR1* mutant background also) carrying pSKiNfeR1 (Fig. 6B). Conversely, *ntrB* and *ntrC* levels in nodules collected 28 days after inoculation of alfalfa plants with SmΔ*nfeR1* were significantly higher than in those elicited by the parent Sm2B3001 strain (Fig. 6C). An additional series of RT-qPCR experiments confirmed the expected pronounced downregulation of *ntrC* in wild-type bacteroids with respect to free-living N stressed bacteria (levels even below those under N surplus), both conditions in which NfeR1 abundance remained high as expected (Fig. S4C). These results indicate that NfeR1 contributes to reach the physiological symbiotic NtrBC output in *S. meliloti*, most likely by counteracting the transcriptional autorepression exerted by NtrC.

### Lack of NfeR1 impairs *S. meliloti* competitiveness under N stress

**Fig 7:**
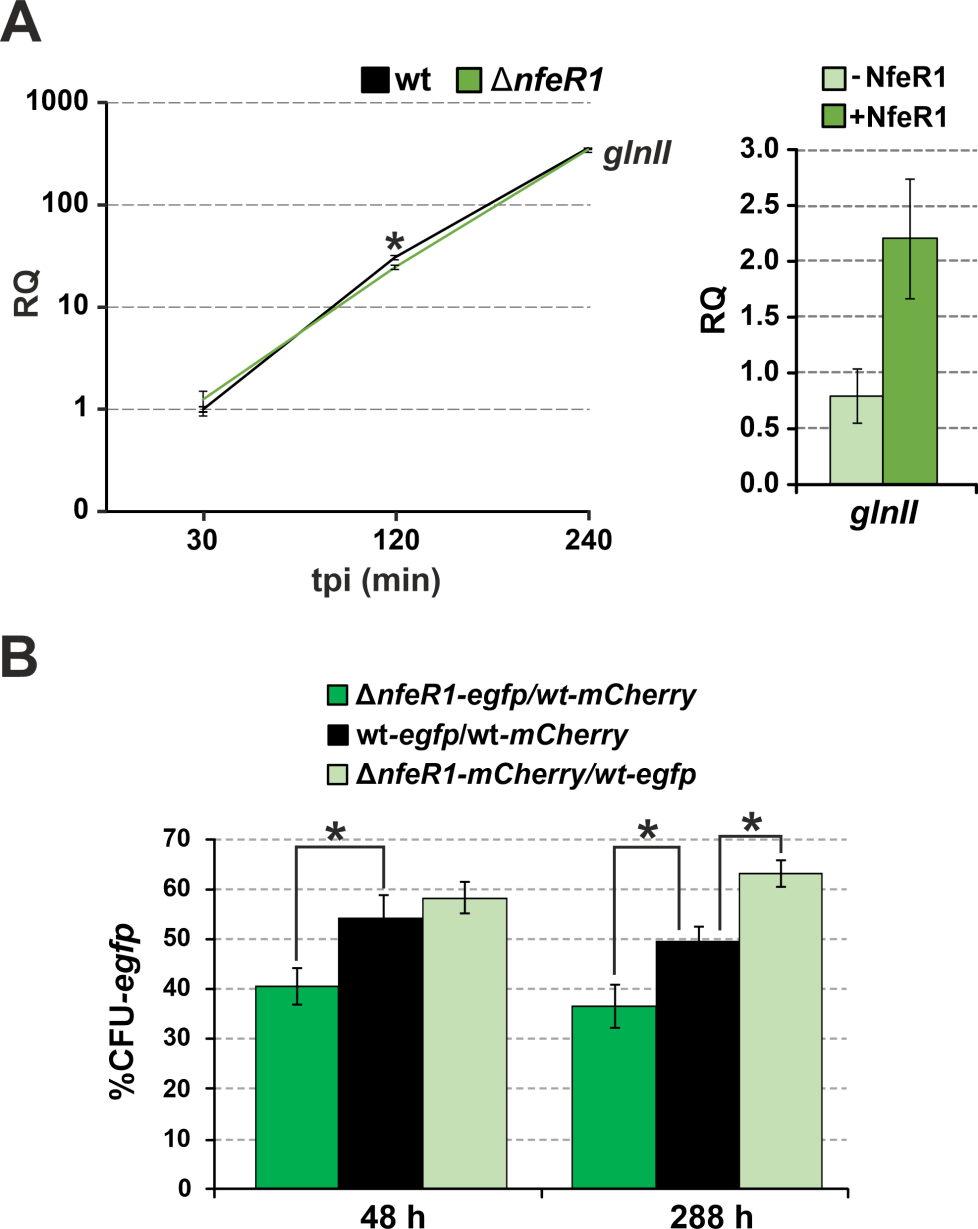
NfeR1 strengthens the NSR in free-living rhizobia. (**A**) RT-qPCR analysis of *glnII* mRNA abundance as NSR marker. RNA was extracted from wild-type Sm2B3001 and derived mutant lacking NfeR1 (left graph) grown in TY to exponential phase, washed in PBS, and cultured in MM for 30 min, 2 h, and 4 h. Two independent cultures were assessed. *glnII* level was also determined in RNA preparations from Sm2020 transformed with pSKiNfeR1 1 h after IPTG induction of NfeR1 expression in MM broth (right graph). Values plotted in the bar graphs are the means and SD of three replicates of three independent cultures. Relative quantification (RQ) values were normalized to *SMc01852* as a constitutive control. (**B**) Lack of NfeR1 attenuates *S. meliloti* competitiveness under N stress. Wild-type Sm2B3001 and SmΔ*nfeR1* bacteria were labeled with both eGFP and mCherry reporters, and MM broth was co-inoculated with different combinations of the reporter bacteria in a 1:1 ratio, as indicated. Three independent cultures of three independent transconjugants were diluted 100-fold in fresh media every 48 h. The percentage of eGFP-labeled bacteria was determined after 48 h and 288 h by counting of green colony forming units (CFUs). Asterisks in the plots indicate significant differences according to the ANOVA test, *P* < 0.05.

Upregulation under N-limiting conditions in culture and the above results anticipate that NfeR1 might assist *S. meliloti* adaptation to N stress. Indeed, we also noticed that the accumulation of *glnII* in SmΔ*nfeR1* parallels that of *ntrC* when N stress was imposed on *S. meliloti* cultures (Fig. 7A; left panel). Pulse ectopic expression of NfeR1 from pSKiNfeR1 in strain Sm2020 most likely restored wild-type NtrC levels and accordingly complemented this molecular phenotype (Fig. 7A; right panel). On the other hand, we previously showed that the lack of NfeR1 does not apparently influence the growth kinetics of *S. meliloti* in MM broth (28). New experiments with Sm2B3001 and SmΔ*nfeR1* strains grown independently in other N-limiting media (e.g., 0.5 mM MM-NH_4_) gave similar results (not shown). Notwithstanding these findings, we further explored the effects of *nfeR1* knockout in competitive growth assays under N starvation. For that, both wild-type and SmΔ*nfeR1* bacteria were labeled with either *eGFP* (green) or *mCherry* (red) by stable genomic integrations of plasmids expressing each reporter constitutively. The N-starving medium MM was then co-inoculated with combinations of differently labeled bacteria in a 1:1 ratio. The number of green and red colonies was recorded, and the ratios were used to estimate the relative abundance of each strain in the co-cultures (Fig. 7B). The ~50% ratio corresponding to the mixture of *eGFP*- and *mCherry*-labeled wild-type cells was thus regarded as equal competence to grow in MM. This ratio significantly decreased 48 h upon co-inoculation of MM with the mixture SmΔ*nfeR1-eGFP*/Sm2B3001-*mCherry* and increased when the inverse labeling combination was assayed. These differences were even more evident upon five consecutive sub-cultivations of the initial cultures. Altogether, these data suggest that NfeR1 helps *S. meliloti* adapt to N stress.

## DISCUSSION

**Fig 8:**
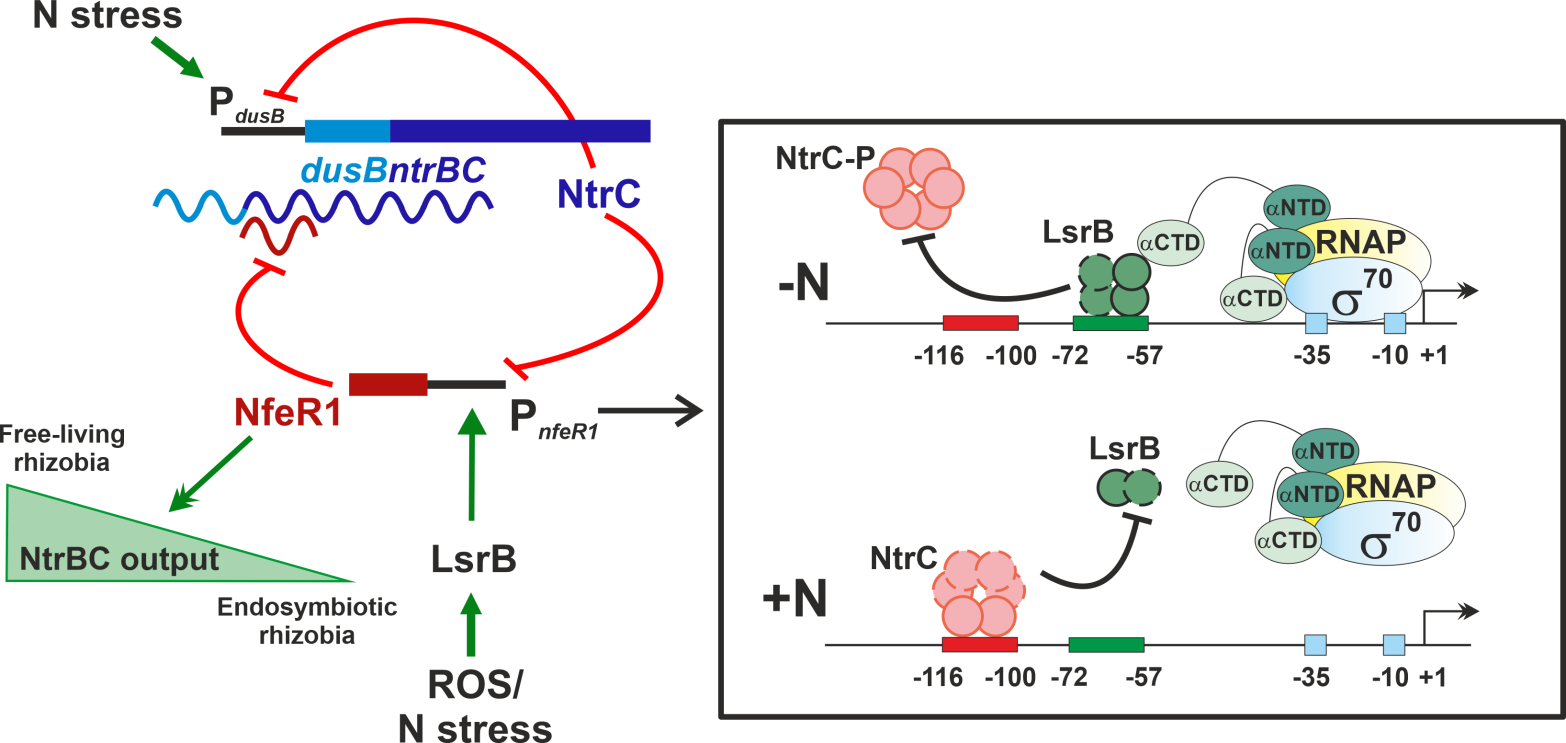
NfeR1 regulation of *S. meliloti* N metabolism. Graphical summary of the results. Straight and curved lines represent genomic DNA regions and RNA transcripts, respectively. Truncated red lines and green arrows (single arrowhead) indicate negative and positive regulation, respectively. Double arrowheads indicate the NfeR1-mediated regulation of the NtrBC output. Inset, proposed model of NfeR1 transcriptional regulation by NtrC and LsrB. Dashed lines in the NtrC and LsrB proteins indicate possible oligomerization. RNAP, RNA polymerase; σ^70^, RpoD; αNTD, N-terminal domain of RNAP α subunit; αCTD, C-terminal domain of RNAP α subunit. Further details in the text.

NfeR1 is a regulatory *trans*-sRNA that influences the symbiotic *S. meliloti* lifestyle pleiotropically. Here, we show that NfeR1 is transcribed from a dual-mode promoter activated by LsrB and repressed by NtrC, which drives sRNA levels to peak under N stress and in N_2_-fixing bacteroids. In turn, NfeR1 feedback regulates the NtrBC TCS by a canonical RNA post-transcriptional silencing mechanism. This mixed double-negative feedback loop tailors NtrBC output to the requirements of *S. meliloti* N metabolism during the symbiotic transition (Fig. 8). To the best of our knowledge, this is the first report describing the RNA-based feedback regulation of NtrBC, the prevalent TCS for N signal transduction in Proteobacteria.

### LsrB and NtrC regulate NfeR1 antagonistically in a N-dependent manner

Bioinformatics, genetics, and biochemical approaches jointly demonstrated that at least two TFs, LsrB and NtrC, bind at distinct nearby sites in P*_nfeR1_* to regulate NfeR1 transcription antagonistically. Co-regulation at complex bacterial promoters such as that of NfeR1 and other sRNAs (e.g., *S. meliloti* MmgR) enables the integration of different environmental signals, thereby linking transcription of individual genes with diverse adaptive traits (37–40). *S. meliloti* LsrB is an oxidative stress-responsive LTTR required for efficient alfalfa nodulation and N_2_ fixation (41–44), which relates NfeR1 with several genuine symbiotic functions, as anticipated by the phenotype of the knockout mutant (28). By contrast, NtrC is largely dispensable at late symbiotic stages (2, 12, 45). In its phosphorylated form, this TF acts as an enhancer-binding protein to activate σ^54^-dependent transcription of N assimilation genes (46). Thus, NtrC is mostly required for rhizobial free-living growth under N-starving conditions. Consistently, our data uncovered an unprecedented role of NfeR1 in the fine-tuning of the *S. meliloti* NSR.

Intracellular NfeR1 levels are primarily adjusted by LsrB functioning as an indispensable activator (likely of Class I) (37), and NtrC as a repressor of transcription. Accordingly, LsrB binding to a motif upstream (positions −72 to −57) of the core promoter elements (σ^70^ −35/–10 hexamers) would enable transcription initiation by recruitment of the RNA polymerase (RNAP) through direct contact with the C-terminal domain of its α subunit (αCTD). In turn, NtrC binding to a site located close upstream (−116 to −100 positions) would preclude LsrB access to the promoter and subsequent transcription by the RNAP. Of note, EMSAs with the apo forms of both recombinant proteins evidenced that NtrC outcompetes LsrB for promoter binding (Fig. 2). Conversely, LsrB was recovered as the major interacting partner of P*_nfeR1_* upon bacterial growth in N starving media, whereas both TFs co-purified when lysates from non-stressed bacteria were used as the protein source in the pull-down assays (Fig. 1). These findings suggest either that this promoter arrangement imposes a steric hindrance that influences binding of the apo and native oligomeric forms of NtrC and LsrB differentially or that oligomerization shifts affinity of the proteins for P*_nfeR1_*. It is known that the reactive oxygen species (ROS) act as co-inducers favoring the formation of LsrB dimers/tetramers *via* intermolecular disulfide bonds between conserved Cys residues located at the C-terminal co-factor binding domain of this regulator (43). ROS can be produced either endogenously as products of *S. meliloti* aerobic respiration, particularly during free-living growth under stress, or by the legume host as a defense response during nodulation (43, 47). On the other hand, NtrC is likely a dimer in solution, and its phosphorylation under N stress promotes the formation of higher-order oligomers (typically hexamers) required for the activation of transcription (46, 48–50). Therefore, ROS-dependent LsrB oligomers would prevent P*_nfeR1_* repression by NtrC *in vivo*, which explains the high NfeR1 transcription and accumulation rates in both endosymbiotic and N-stressed free-living *S. meliloti* bacteria.

NtrC activity as a transcriptional repressor in bacteria is still scarcely recognized (14, 51). The NtrC-binding motif at P*_nfeR1_* is also identifiable in NtrC-activated promoters such as that of *glnII* in several rhizobia (33, 52). Thus, besides phosphorylation, transcriptional activation by NtrC requires co-occurrence of the σ^54^ signature and an enhancer region upstream of the NtrC-binding site (46), which are both absent in P*_nfeR1_*. Collectively, our data evidenced that NtrC can repress P*_nfeR1_* irrespective of its phosphorylation and oligomerization states, that is, in both N-sufficient and -limited media, and in the absence of NtrB (Fig. 3 and 4). Nonetheless, expression profiling of NfeR1 revealed that repression of P*_nfeR1_* by NtrC prevails over LsrB-mediated activation under N surplus in complete or defined media, in which NtrC remains mostly as a dimeric dephosphorylated protein. It is also noteworthy that a mutant lacking the histidine-kinase NtrB does not have a growth defect in N-limiting media as noticeable as that of the *ntrC* mutant (not shown), further suggesting that the function of this largely considered inactive form of NtrC is underestimated in *S. meliloti*, even in the frame of the NSR. Overall, our data show that P*_nfeR1_* architecture supports a dynamic output in response to N fluctuations.

### Feedback regulation by NfeR1 amends NtrBC output

The function of bacterial RNA regulators in environmental adaptation is further delineated by their target mRNAs, typically multiple for a single *trans*-sRNA (53). The regulatory potential of NfeR1 relies on three functionally redundant aSD motifs, similar but not identical to those of the sibling AbcR1 and AbcR2 sRNAs functionally characterized in *S. meliloti* and related α-proteobacteria (28, 31). Consequently, the AbcR1/2 regulon, mostly consisting of transport and metabolic mRNAs, is predicted to overlap largely with that of NfeR1. Co-activation of AbcR1 and NfeR1 transcription by LsrB further hints at akin functions of both sRNAs based on pervasive downstream regulation of *S. meliloti* metabolism. However, the markedly different AbcR1 and NfeR1 loss-of-function phenotypes envisaged relevant functional specificities (28, 30, 31). Feedback silencing of the *ntrBC* mRNA is a major distinctive feature specifically placing NfeR1 at the core of the NSR in *S. meliloti*. Our findings thus illustrate the functional versatility of two sRNAs with comparable regulatory abilities.

Genetic reporter assays based on ectopic co-expression of the regulatory pair NfeR1-*ntrB*, uncoupled from the endogenous NSR, support a conventional RNA silencing mechanism involving primary occlusion of the *ntrB* ribosome binding site by base-pairing to the NfeR1 aSD seeds, and plausible subsequent mRNA decay. Consistently, as part of the polycistronic *dusBntrBC* mRNA, downregulation of *ntrB* upon induced NfeR1 (over)expression results in reduced NtrB and NtrC levels as revealed by these assays. Unlike AbcR1/2, NfeR1 is a representative of the large fraction of *S. meliloti* Hfq-independent *trans*-sRNAs (54). The identity of the RNA chaperone(s), if any, assisting the *ntrB*-NfeR1 interaction thus remains as an open question regarding the NfeR1 activity mechanism.

Post-transcriptional feedback silencing of *ntrBC* by NfeR1 is a novel mode of control of this TCS in rhizobia, added to the known transcriptional autorepression of the *dusBntrBC* operon by NtrC (10, 15, 17). Negative feedback occurs ubiquitously in natural eukaryotic and prokaryotic gene control systems as a major mechanism to reduce output variations in response to external input signals (18, 55–57). Protein-based autorepression typically yields a sigmoidally shaped input-output response, allowing only rough tuning of the output with external signaling. By contrast, regulatory sRNAs produce faster and linear responses, providing more tightly controlled feedback (58, 59). Our data show that NfeR1 does influence the output of the system, that is*, ntrBC* levels, but differently in free-living and symbiotic rhizobia (Fig. 6). In bacteria cultured under N starvation, that is*,* the peak of NtrBC (Fig. S4A), lack of NfeR1 resulted in transient downregulation of *ntrBC*. Conversely, within nodules, that is*,* lowest *ntrBC* levels (Fig. S4C), the effect of the *nfeR1* knockout was the opposite. Therefore, in the context of the endogenous regulation of *S. meliloti* N metabolism, NfeR1-mediated silencing of *ntrBC* would reduce the effective feedback strength of the NtrC autorepressor to achieve an accurate and uniform output, that is, constant physiological *ntrBC* levels.

Bacterial lifestyle drives the evolution of complex network architectures featured by frequently redundant regulatory motifs layered onto core adaptive systems such as TCSs. These systems are so resilient that can retain function despite their recurring regulatory network motifs are individually disabled (60–64). Consistently with this notion, we noticed a discrete but reliable impact of *nfeR1* knockout in the abundance of major effectors of the NSR (e.g*., glnII*) and *S. meliloti* growth under N stress (Fig. 7). Furthermore, the latter was only evident when the mutant competed for growth with the wild-type strain. We, therefore, conclude that NfeR1 confers *S. meliloti* an advantage to efficiently compete for colonizing N-deficient environments.

### Broad impact of the NtrBC-NfeR1 regulatory loop in symbiosis

N-signaling operates in rhizobia and their legume hosts throughout the symbiotic interaction (2). High concentrations of combined N in soil inhibit nodule organogenesis, which is primarily controlled by the host plant. In response to N availability, *S. meliloti* NtrBC not only adjusts N assimilation accordingly but also the levels of Nod factors synthesized in the presence of the root-exuded flavonoid luteolin. The latter occurs by NtrBC-mediated transcriptional activation of the *nodD3* regulatory gene, which is likely a specific feature of *nod* gene regulation in *S. meliloti* and the broad host-rage *Rhizobium* sp. NGR234 (4, 65). Tight feedback control of NtrBC by NfeR1 would thus endow *S. meliloti* with the symbiotic competence to efficiently cope with the N stress demanded by the plant at the onset of nodulation. This explains further the reported impact of *nfeR1* knockout in *S. meliloti* competitiveness for nodule formation (28). However, in endosymbiotic bacteroids, N_2_ fixation is uncoupled from the bacterial NSR so that the ammonia generated in the process is fully transferred to the plant (2, 12). We previously showed that the *S. meliloti nfeR1* mutant is not as efficient as the wild-type strain in promoting plant growth. Nonetheless, a lack of NfeR1 delays but does not apparently compromise terminal bacteroid differentiation and symbiotic N_2_ fixation (28). At this late symbiotic stage, NfeR1-mediated post-transcriptional downregulation of *ntrBC* outweighs autorepression by NtrC, which might contribute to silencing N assimilation to the benefit of the plant.

Both the P*_nfeR1_* architecture and *ntrB* targeting by NfeR1 are predicted to be conserved across α-rhizobia. Thus, this unprecedented NtrBC-NfeR1 double-negative feedback loop likely operates ubiquitously in these bacteria to adjust N metabolism to the demands of an efficient symbiosis with legume plants.

## MATERIALS AND METHODS

### Bacterial strains, plasmids, and growth conditions

Bacterial strains and plasmids used in this work, with their relevant characteristics, are listed in Table S1. *Escherichia coli* strains were routinely grown in lysogeny broth (LB) medium at 37°C (66), and rhizobia in either complex tryptone-yeast (TY) or defined mannitol/glutamate MM media at 30°C (67, 68). To test the effect of shifts in N metabolism on NfeR1 accumulation, L-glutamate (6.5 mM) was replaced by NH_4_Cl (10 or 0.5 mM) or KNO_3_ (10, 0.5, or 0.05 mM) in the standard MM. The osmotic upshift was imposed by adding 400 mM NaCl to exponential cultures. When required, growth media were supplemented with the appropriate antibiotic(s) (µg/mL): streptomycin (Sm) 480, tetracycline (Tc) 10, gentamycin (Gm) and kanamycin (Km) 50 for *E. coli,* and 180 for *S. meliloti*. For growth in liquid media, the antibiotic concentration was reduced to 50%.

### DNA oligonucleotides

Sequences of the oligonucleotides used for cloning, RT-qPCR and as probe in Northern hybridizations are provided in Table S2.

### DNA pulldown assays

The BtnPC14Fw/P14C2Rv primer pair was used for the amplification of Btn-P*_nfeR1_* from genomic DNA. The promoter fragment lacking the conserved motif was generated by a two-step PCR on genomic DNA. The first amplification round with the primer pairs BtnPC14Fw/PC14FusRv and PC14FusFw/P14C2Rv yielded overlapping fragments flanking this motif. The second, with BtnPC14Fw/P14C2Rv, generated the full-length Btn-P*_nfeR1_*_Δ_ DNA probe. Both Btn-P*_nfeR1_* and Btn-P*_nfeR1_*_Δ_ DNA fragments were concentrated and purified by phenol-chloroform extraction followed by ethanol precipitation. The DNA-chromatography pull-down assay was adapted from a previously published protocol (69). An amount of 200 µL of streptavidin resin (GenScript, Cat. No. L0053) was washed four times in wash buffer (20 mM Tris-HCl pH 8, 1 mM EDTA, 150 mM NaCl), resuspended in 0.6 mL of wash buffer containing 40 µg of biotinylated DNA and incubated at 4°C for 1 h. Cells equivalent to 400 OD_600_ were harvested, washed once with 0.1% sarcosyl in TE buffer (100 mM Tris-HCl, 10 mM EDTA, pH 8), and frozen in liquid N_2_. Then, cells were resuspended in 4 mL of protein binding buffer (20 mM Tris-HCl buffer pH 8, 1 mM EDTA, 10 mM HEPES, 10% glycerol, 100 mM NaCl, and 0.05% Triton X-100) supplemented with cOmplete Protease Inhibitor Cocktail (Roche) and then lysed by three consecutive passes (1,000 psi) through a French press. After centrifugation at 12,000× *g* and 4°C for 10 min, the supernatant was added to the streptavidin resin and DNA baits, previously washed in 500 µL of protein binding buffer three times and supplemented with bovine serum albumin (BSA) (5 µg/mL). The mixture was incubated at 4°C for 16 h in a BellyDancer Shacker. After centrifugation at 8,200× *g* and 4°C for 1 min, the supernatant was removed leaving 0.8 mL of buffer to resuspend the pellet to be loaded into a SigmaPrep spin column (Sigma) previously washed twice with protein binding buffer. Then, the resin was washed three times with protein binding buffer supplemented with cOmplete Protease Inhibitor Cocktail and 5 µg/mL BSA. Finally, the resin was incubated for 10 min with 120 µL of elution buffer (50 mM Tris-HCl pH 8) containing increasing concentrations of NaCl upon centrifugation at 8,200× *g* for 2 min. The eluted fractions were run in a 15% SDS-PAGE and proteins were visualized using the Silver Stain kit (BioRad). To monitor the presence of DNA baits, DNA was released from streptavidin resin by adding 60 µL of 8 M guanidine·HCl (pH 1.5), centrifugation and stabilization with 60 µL of 1 M Tris-HCl pH 8.

### Liquid chromatography-mass spectrometry

For protein identification, the different eluted fractions corresponding to each DNA bait and culture condition were mixed and concentrated to be loaded in a 4% SDS-PAGE run at 6 mA. Protein samples were further run for 10 min in running buffer (0.124 M Tris-HCl, 1.252 M glycine, 5% [wt/vol] SDS) and then gel was stained using Coomassie Blue to isolate gel lanes. Alternatively, protein samples were run in a 10% SDS-PAGE at 150 V and, after Coomassie Blue staining, major protein bands were isolated. MS analyses were performed at the Proteomics Service from Instituto de Parasitología y Biomedicina “López-Neyra” (CSIC, Granada).

### Construction of *S. meliloti* mutants

Knockout mutants were generated using the suicide plasmid pK18*mobsacB* as previously described (70, 71). SmΔ*ntrC* and SmΔ*ntrB* were generated in Sm2B3001 by a markerless in-frame deletion of the *ntrC* and *ntrB* CDSs using pK18Δ*ntrC* and pK18Δ*ntrB,* respectively. To construct pK18Δ*ntrC,* 792-nt and 749-nt DNA fragments flanking the *ntrC* ORF were amplified from genomic DNA with the BamHIntrBFw/ATGXbaIRv and XbaITGAFw/ntrYHindIIIRv primer pairs. PCR fragments were digested with *Bam*HI/*Xba*I and *Xba*I/*Hind*III, respectively, and ligated to the pK18*mobsacB Bam*HI and *Hind*III restriction sites, leading to the insertion of the fragments in tandem via their common *Xba*I site. Similarly, pK18Δ*ntrB* was generated by amplification of 816-nt and 801-nt DNA fragments from genomic DNA with the BamHIntrBupFw/ntrBupXbaIRv and XbaIntrBdownFw/ntrBdownHindIIIRv primer pairs, subsequent digestion with *Bam*HI/*Xba*I and *Xba*I/*Hin*dIII and ligation between the pK18*mobsacB Bam*HI and *Hin*dIII restriction sites. Plasmids were mobilized to the parent strains by biparental mattings (72).

### Fluorescence reporter assays

The transcriptional fusions reporting promoter activity were first generated in the promoterless vector pBB::*eGFP* (28). The full-length NfeR1 promoter (P*_nfeR1-213_*) was amplified using P14C2EcoRIFw and P14C2XbaIRv primers from genomic DNA, digested with *Eco*RI/*Xba*I, and cloned into pBB::*eGFP* to generate pBBP*_nfeR1-213_::eGFP*. The *glnII* (P*_glnII_*), *dusB* (P*_dusB_*), and *ntrC* (P_ntrC*_) promoters were amplified from genomic DNA with the primer pairs HindIIIPglnII-400F/XbaImTSSglnIIR, HindIIIPdusB/PdusBXbaIRv, and mTSSntrC300up/PntrCXbaIRv, respectively. The PCR products were digested with *Hin*dIII/*Xba*I and cloned into pBB::*eGFP* yielding pBBP*_glnII_::eGFP,* pBBP*_dusB_::eGFP,* and pBBP_ntrC*_::eGFP. The 100-nt long promoter P_nfeR1-100*_ containing point mutations at the LsrB binding site was generated by annealing the oligonucleotides EcoRIPc14mutFw/XhoIPc14mutRv and further insertion of the resulting product between the *Eco*RI and *Xho*I restriction sites in pBB::*eGFP,* yielding pBBP_nfeR1-100*_::eGFP. The 213-nt long promoter P_nfeR1-213*_ containing point mutations at the NtrC binding site was generated by a two-step PCR on pBBP*_nfeR1-213_::eGFP* vector. The first amplification round with the primer pairs SR_Fw/NtrCbsRv and NtrCbsFw/GFP_Rv yielded overlapping fragments for a second amplification round with SR_Fw/GFP_Rv. This product was restricted with *Eco*RI and *Xba*I and cloned into pBB::*eGFP* for generation of pBBP_nfeR1-213*_::eGFP. The different promoter-reporter fusions were next inserted into the single-copy plasmid pABCa by amplification with avrIISRFw and avrIIGFPRv primers using pBB*_PnfeR1-213_::eGFP*, pBB_PnfeR1-213*_::eGFP, pBBP*_nfeR1-100_::eGFP,* and pBBP*_nfeR1-40_::eGFP* as DNA templates. Then, PCR products were digested with *Avr*II and cloned into pABCa.

The translational reporter fusion of *ntrB* to *eGFP* was generated in plasmid pR-eGFP (30). For this, the translation initiation region of *ntrB* (positions −143 to +48 relative to the start codon) was amplified with the ntrBF/ntrBR primer pair. The resulting PCR product was digested with *Bam*HI/*Nhe*I and cloned into pR-eGFP to yield pR*ntrB::eGFP*. The reporter plasmid was transferred by biparental conjugation to Sm2020 harboring plasmids expressing either wild-type NfeR1 or the NfeR1abc variant (28). Double transconjugants were grown to the exponential phase (OD_600_ of 0.2 to 0.3), divided into untreated and 0.5 mM-IPTG-treated cultures, and incubated for 24 h.

OD_600_ and fluorescence (excitation 485 nm and emission 520 nm) from bacteria transformed with transcriptional or translational fusion to eGFP were measured in a Thermo Scientific Varioskan LUX multimode microplate reader. Fluorescence values were normalized to the culture OD_600_ and the medium fluorescence background.

### Construction of plasmids for induced *ntrC* expression

For the IPTG-induced expression of the *ntrC* gene, the NtrC CDS was amplified from genomic DNA using the NtrC_Fw_NdeI/ NtrC_Rv_HindIII primer pair. The PCR product was digested with *Nde*I and *Hin*dIII and inserted into pSRKKm, yielding pSRK-*ntrC*.

### Purification of proteins

A His-tagged LsrB encoded in p16LsrB was produced and purified as described (73). The NtrC coding sequence was PCR amplified from genomic DNA as described above and cloned into vector pET-29a (Novagen) between the *Nde*I/*Hin*dIII restriction sites, yielding p29NtrC for native NtrC overexpression. p16LsrB and p29NtrC plasmids were mobilized to *E. coli* BL21(DE3) by electroporation. NtrC purification was performed as previously described (74).

### EMSAs and DNA footprinting

The P14C2Fw/P14C2Rv and P14C2EcoRIFw/P14C2XbaIRv primer pairs were used to amplify P*_nfeR1-100_/*P_nfeR1-100*_ and P*_nfeR1-213_/*P_nfeR1-213*_, respectively, using pBB::*eGFP* carrying promoter fragments of different length as DNA templates. A deleted version of the *nfeR1* promoter (P*_nfeR1-40_*) was generated by annealing oligonucleotides P14C2_54 and P14C2_54i. The DNA fragments were purified from agarose gels and then labeled at their 5′-ends with [γ−32P]-dATP and T4 polynucleotide kinase. Binding reactions were performed with 1 nM radiolabeled probes in the absence or presence of purified LsrB or NtrC in 20 µL of STAD [25 mM Tris-acetate pH 8.0, 8 mM Mg-acetate, 10 mM KCl, 3.5% (wt/vol) polyethylene glycol-8000 and 1 mM DTT] supplemented with 15 µg/mL of poly(dI-dC) and 200 µg/mL of BSA. The reaction mixtures were incubated for 30 min at 4°C, and samples were run on 4.5% (wt/vol) native polyacrylamide gels (4.5% acrylamide/bis-acrylamide 29:1 [VWR] 25 mM Tris, 200 mM glycine, 1% [wt/vol] APS and 0.5 µL per mL TEMED) at 4°C for 3 h in Tris-glycine buffer (25 mM Tris, 200 mM glycine). To test protein competition, DNA was incubated with one protein for 30 min before the addition of different concentrations of the other protein. The results were analyzed with Personal FX equipment and the Quantity One software (Bio-Rad).

For DNA footprinting, P*_nfeR1-213_* was amplified with P14C2EcoRIFw and 5′ end-labeled P14C2XbaIRv. Binding reactions were performed with 20 nM radiolabeled probes in the absence or presence of purified NtrC before being treated with 100 µL of 1:80,000 DNase I dilution at 30°C for 2 min. Then, binding reaction mixtures were concentrated and purified by phenol-chloroform extraction followed by ethanol precipitation and resuspended in 6 µL of TE (10 nM Tris-HCl and 0.1 mM EDTA, pH 8) and 3 µL of loading dye. Equal amounts of DNA (5,000 cpm) were loaded in 6% (wt/vol) denaturing polyacrylamide gel (6% acrylamide/bis-acrylamide 29:1 [VWR], 7M urea [Sigma], 89 mM Tris-HCl pH 8, 89 mM boric acid and 2 mM EDTA, 1% [wt/vol] APS, and 0.5 µL per ml TEMED). The Thermo Sequenase Cycle sequencing kit (Applied Biosystems) was used for the generation of a sequencing ladder from the DNA probe. The results were analyzed with Personal FX equipment and Quantity One software (Bio-Rad).

### Northern blot hybridization

Total RNA was isolated from bacterial pellets by acid phenol/chloroform extraction as previously described (75). For Northern analysis, RNA samples (typically 10–20 μg) were subjected to electrophoresis on 6% polyacrylamide/7 M urea gels in TBE Tris-borate-EDTA) at ~30 mA and electro-transferred to nylon membranes, which were subsequently probed with 5′-end radiolabeled 25mer oligonucleotides specific for the NfeR1 and 5S RNA following previously described protocols (76).

### RT-qPCR

RNA samples were additionally treated with Invitrogen DNase TURBO for 1 h at 37°C and further cleaned up with the RNeasy Mini Kit (Qiagen) following the manufacturer’s guidelines. To improve retention of the sRNA fraction, RNA samples are loaded onto the columns mixed with 7 volumes of 100% ethanol. cDNA was synthesized with the Takara Prime Script RT Master Mix (Perfect Real Time) using 1 µg of total RNA. RT-qPCR was carried out in a QuantStudio 3 (Thermo Fisher Scientific) with the Takara TB Green Premix ExTaqII (Tli RNaseH Plus) using 0.5 µL of cDNA following the manufacturer’s instructions. The ratios of transcript abundance were calculated as the ΔΔCT mean average of two or three independent RNA extracts. The *S. meliloti* constitutively expressing gene *SMc01852* was used to normalize gene expression (77). Control reactions without reverse transcriptase (–RT) in the RNA samples were simultaneously performed to confirm absence of DNA contamination.

### Tagging and production of proteins with 3×FLAG

The pR_FLAG vector was constructed by annealing oligonucleotides NheISphIFlagF/EcoRIFlagR and cloned into vector pR-EGFP between the *Nhe*I/*Eco*RI restriction sites. This vector was used to tag NtrB and NtrC at their C-termini with three consecutive units of the FLAG epitope (3×FLAG). For generation of pR*ntrB^FLAG^* and pR*ntrBntrC^FLAG^*, the NtrB and NtrB-NtrC CDSs (excluding the stop codon) along with the *ntrB* translation initiation region were amplified using ntrBF/ntrBNheI and ntrBF/ntrCNheI primer pairs, respectively. These PCR products were digested with *Bam*HI/*Nhe*I and cloned into pR_FLAG, where the tagged protein is encoded downstream of the P*_syn_* constitutive promoter. To generate pR*dusBntrBntrC^FLAG^* and pR*ntrC^FLAG^*, the full-length operon (including a 243-nt long promoter region) and the NtrC CDS, including a 300-nt upstream region to its putative TSS, were amplified with HindIIIPdusB/ntrCNheI and mTSSntrC300up/ntrCNheI primer pairs. The DNA fragments were digested with *Hin*dIII/*Nhe*I and cloned into pR_FLAG.

### Protein immunoblot

For Western blot, aliquots equivalent to 0.05 OD_600_ of cells were denatured by heating at 95°C for 5 min, resolved in 10% SDS-PAGE, and blotted onto a polyvinylidene difluoride membrane (P 0.45, Amersham). Membranes were probed with a monoclonal anti-FLAG antibody (Sigma F7425; 1:5,000) as reported (73). Blots were developed by incubation for 5 min in blotting detection reagent (ECL, Amersham) and signals were detected with a ChemiDoc system (BioRad). The intensity of lanes was quantified using ImageJ software (78).

### Competitive growth assay

The estimation of the relative fitness of the deletion mutant SmΔ*nfeR1* was performed as previously described (63). For that, wild-type and SmΔ*nfeR1* strains were labeled with *mCherry* or *eGFP* by single genomic integration of either plasmid pKOSm or pKOSe. Strains were individually grown in TY media for 48 h and then bacteria were diluted in MM fresh media to OD_600_ of 0.005 and mixed at a ratio of 1:1 in a final volume of 4 mL. Every 48 h of incubation, the mixed population was diluted 100-fold in fresh media a further cultured. The procedure was repeated for 6 days (288 h) for each starting culture. The first and last cultures were diluted and plated in TY plates for determination of the percentage of *eGFP*- and *mCherry*-labeled CFUs using a Leica M165FC stereomicroscope equipped with ET GFP2 (excitation band pass 480/40 nm and emission band pass 510 nm) and ET TXR LP (excitation band pass 560/40 nm and emission band pass 610 nm) filters. Image acquisition and adjustment were done with Leica Application Suite EZ 3.4.0 software.

### Plant assay

*Medicago sativa* L. ‘Aragón’ seeds were surface sterilized and germinated on 1.5% water agar plates in the dark at 28°C for 24 h, and transferred to test tubes containing 10 mL of nitrogen-free nutrient solution (Rigaud and Puppo) as previously described (79). Seedlings were inoculated with 1 mL of a 10^6^ bacterial suspension of either the wild-type Sm2B3001, Sm∆*lsrB* or Sm∆*ntrC* strains carrying pBB*_PnfeR1-213_::eGFP*. GFP fluorescence during bacterial root hair colonization and infection thread formation was observed at 6- and 9 days post-inoculation, respectively. Roots were analyzed by laser scanning confocal microscopy with a Nikon C-1 microscope using 488 nm argon laser excitation. Root nodules were harvested 28 days after inoculation of plants. Bacteroids and total RNA from nodules were isolated as previously described (76, 80). Bacteroids were visualized under an epifluorescence microscopy using a Leica DMI6000B microscope equipped with filter set ET GFP (excitation band pass 470/40 nm, beam splitter 500 nm, and emission band pass 525/50 nm). Image acquisition and adjustment were done with Leica Application Suite EZ 3.4.0 software.

### Bioinformatic tools

Promoter sequence alignments were generated with ClustalW implemented in BioEdit (https://bioedit.software.informer.com/7.2/). Logos of consensus sequence motifs were generated at http://weblogo.berkeley.edu/logo.cgi. sRNA-mRNA base-pairing interactions were predicted with IntaRNA and CopraRNA (http://rna.informatik.uni-freiburg.de/).

## SUPPLEMENTAL MATERIAL

Fig. S1 mbio.02003-23-s0001.tif Alignment of NfeR1 promoter sequences.

Fig. S2 mbio.02003-23-s0002.tif NtrC footprinting.

Fig. S3 mbio.02003-23-s0003.tif Transcriptional regulation of NfeR1 in symbiosis.

Fig. S4 mbio.02003-23-s0004.tif Transcriptional regulation of NtrC.

Supplemental legends mbio.02003-23-s0005.docx Legends to Fig. S1 to S4 and Tables S1 and S2.

Table S1 mbio.02003-23-s0006.docx Bacterial strains and plasmids.

Table S2 mbio.02003-23-s0007.docx Oligonucleotides.

ASM does not own the copyrights to Supplemental Material that may be linked to, or accessed through, an article. The authors have granted ASM a non-exclusive, world-wide license to publish the Supplemental Material files. Please contact the corresponding author directly for reuse.
