# Supplementary figures and images for: A double-negative feedback loop between NtrBC and a small RNA rewires nitrogen metabolism in legume symbionts
Source: mBio. 2023 Oct 18;14(6):e02003-23. doi: 10.1128/mbio.02003-23 (PMC10746234; doi:10.1128/mbio.02003-23)

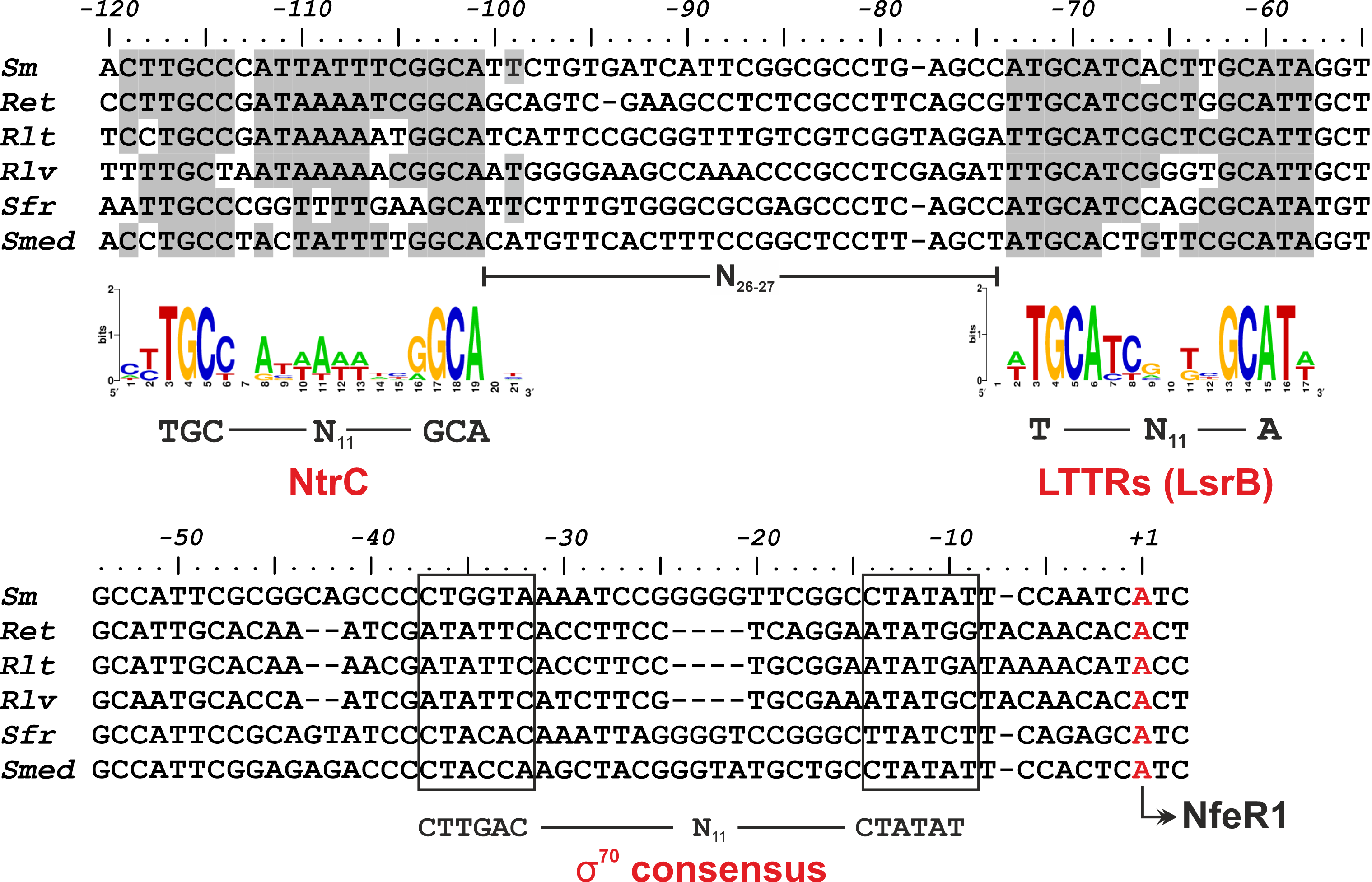

Supplement: Fig. S1 — Alignment of NfeR1 promoter sequences. [file mbio.02003-23-s0001.tif]

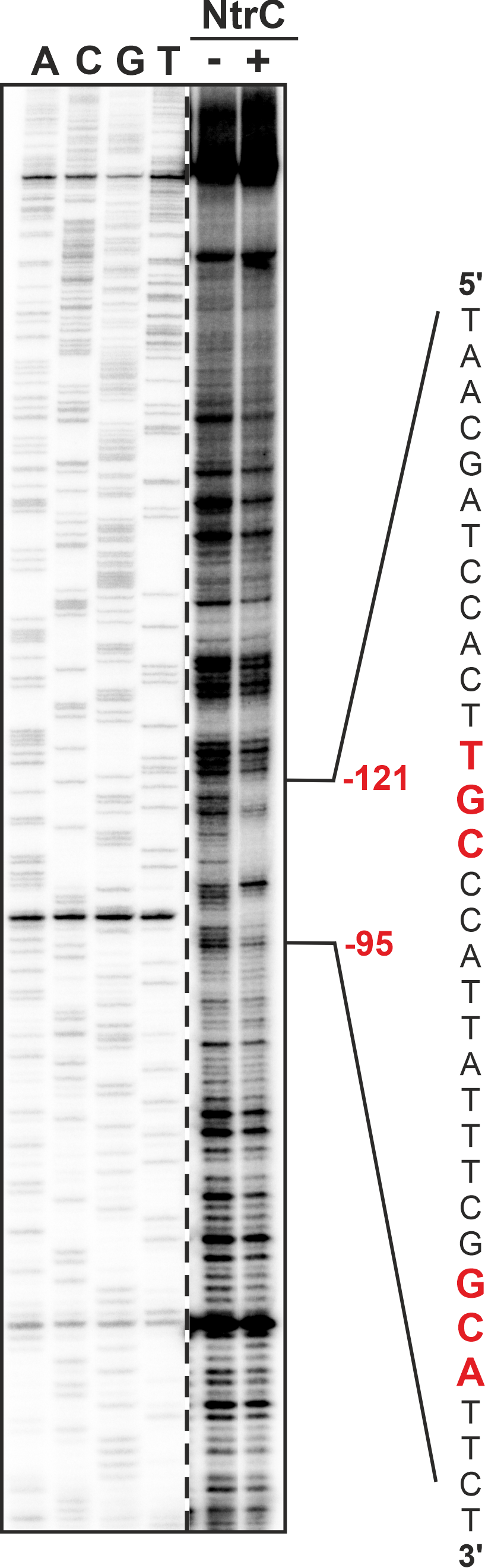

Supplement: Fig. S2 — NtrC footprinting. [file mbio.02003-23-s0002.tif]

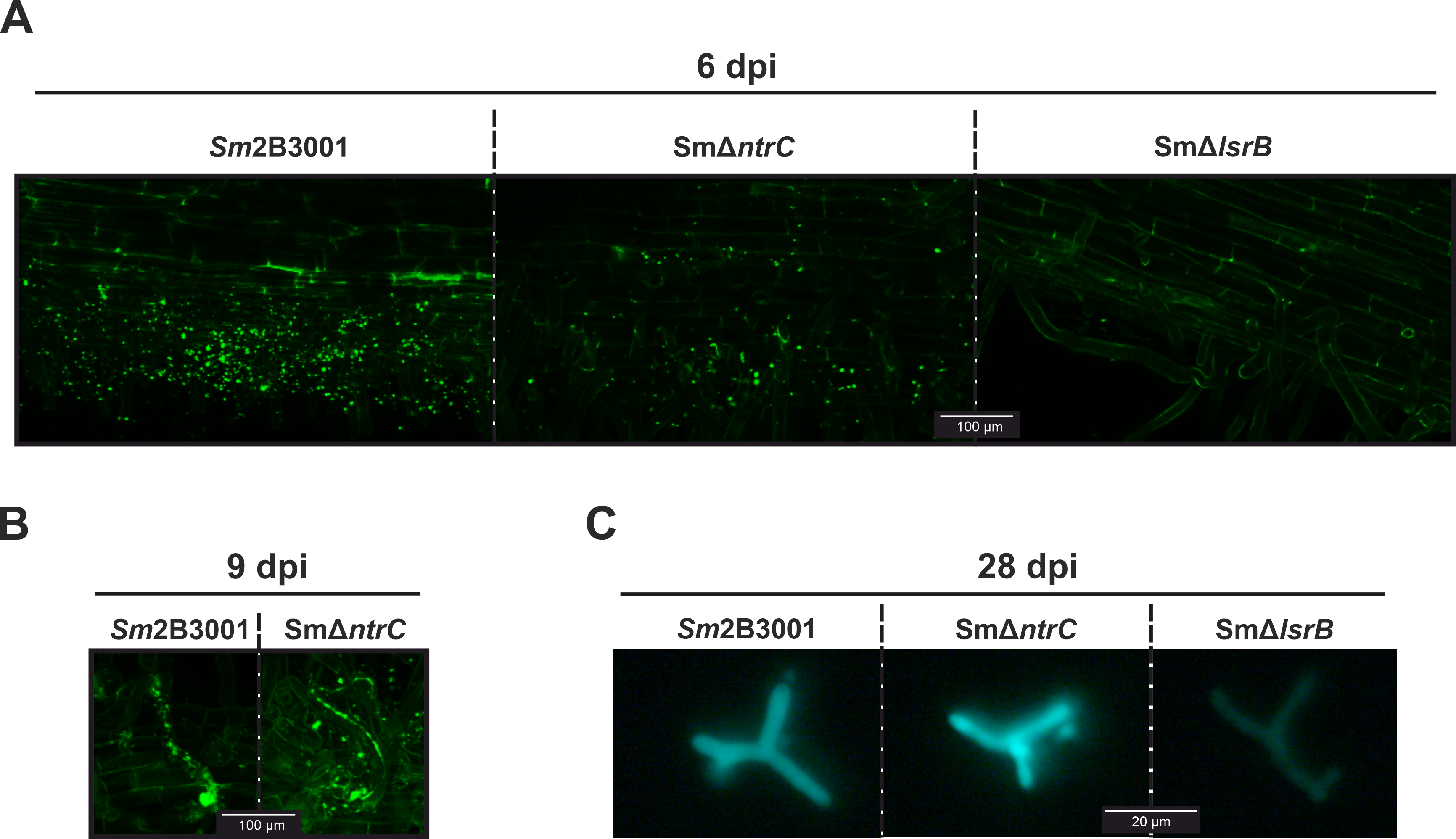

Supplement: Fig. S3 — Transcriptional regulation of NfeR1 in symbiosis. [file mbio.02003-23-s0003.tif]

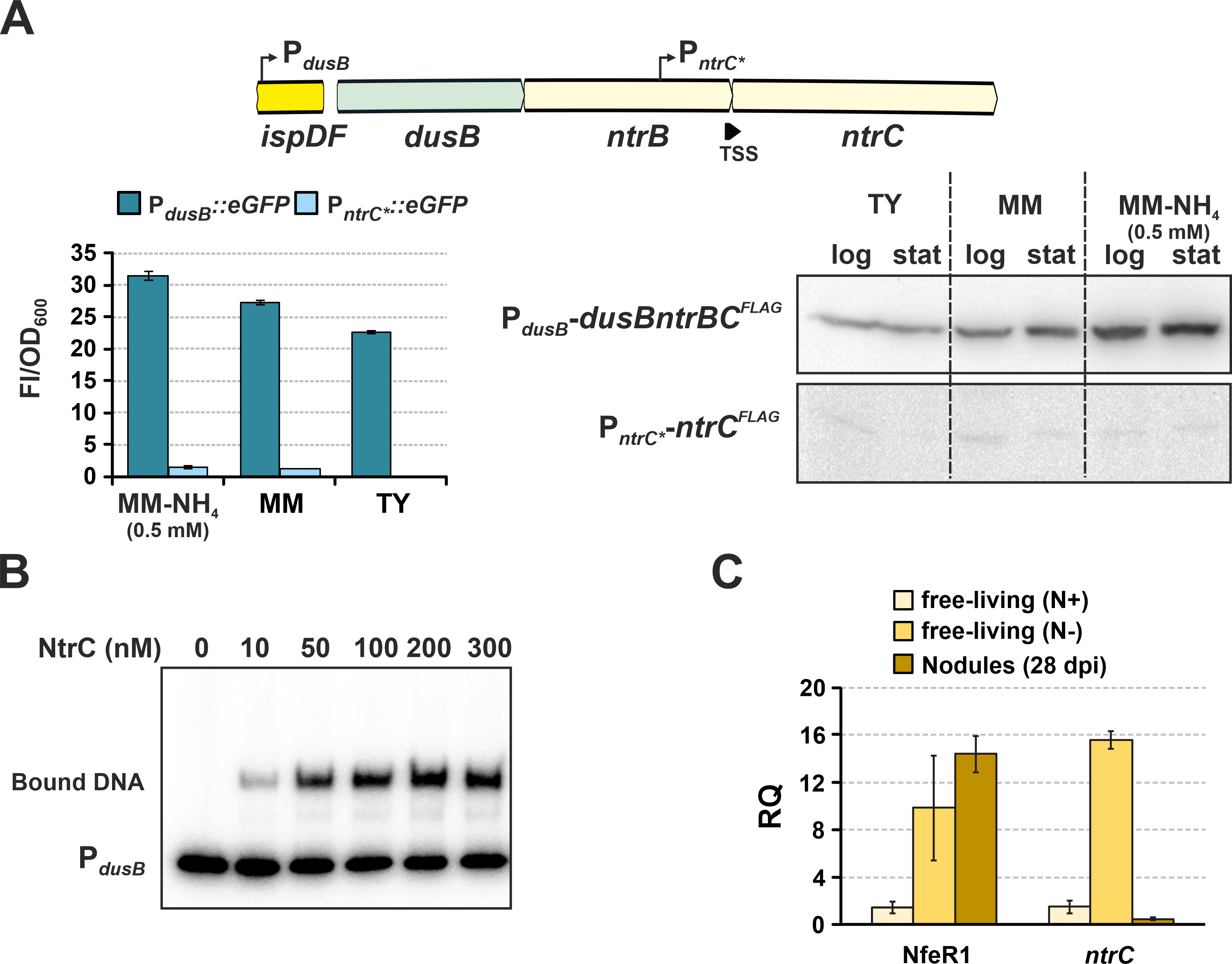

Supplement: Fig. S4 — Transcriptional regulation of NtrC. [file mbio.02003-23-s0004.tif]
