# Supplementary material for: A double-negative feedback loop between NtrBC and a small RNA rewires nitrogen metabolism in legume symbionts
Source: mBio. 2023 Oct 18;14(6):e02003-23. doi: 10.1128/mbio.02003-23 (PMC10746234; doi:10.1128/mbio.02003-23)
Supplement: Supplemental legends — Legends to Fig. S1 to S4 and Tables S1 and S2. [file mbio.02003-23-s0005.docx]

**SUPPLEMENTAL MATERIAL**

**FIG. S1.** Multiple sequence alignment of the promoter regions of NfeR1 homologs in α-rhizobia. Consensus sequences of the conserved motifs are indicated below the alignments. *Sm*; *Sinorhizobium meliloti* Sm1021; *Ret*, *Rhizobium etli* CIAT652; *Rltr*, *R. leguminosarum* bv. *trifolii* WSM2304; *Rlv*, *R leguminosarum* bv. *viciae* 3841; *Sfr,* *S. fredii* HH103; *Smed*, *S. medicae* WSM419.

**FIG. S2.** NtrC footprinting. P*_nfeR1-213_*, 5′ end-labeled on the bottom strand, was incubated without (-) and with NtrC (1 μM; +). After partial digestion with DNase I, the reactions were subjected to urea-PAGE. The nucleotide sequence protected by NtrC, as inferred by the sequencing ladder from the P*_nfeR1-213_* fragment (A, C, G, T lanes), is indicated to the right.

**FIG. S3.** Transcriptional regulation of NfeR1 in symbiosis. (A) Fluorescence derived from P*_nfeR1-213_::eGFP* (pBB::*eGFP* plasmid) at early symbiotic stages. Shown are representative confocal microscopy images of alfalfa roots 6 days after inoculation with wild-type, NtrC and LsrB mutants (Sm2B3001, SmΔ*ntrC* and SmΔ*lsrB*, respectively) harboring the reporter plasmid. (B) NfeR1 promoter activity in the infection threads. Fluorescence from P*_nfeR1-213_::eGFP* was visualized by confocal microscopy 9 days after plants inoculation with the reporter Sm2B3001 and SmΔ*ntrC* strains. (C) NtrC- and LsrB-dependent regulation of NfeR1 in mature bacteroids. Shown are representative epifluorescence microscopy images of bacteroids isolated from 28 days-old nodules elicited by wild-type, SmΔ*ntrC* and SmΔ*lsrB* reporter strains.

**FIG. S4.** Transcriptional regulation of NtrC. (A) Analysis of the *ntrC* promoter activity. Diagram of the *S. meliloti* *ntrC* genomic region indicating also the annotated TSS and the putative promoters (P*_dusB_* and P*_ntrC*_*). Activity of the transcriptional fusions of P*_dusB_* and P*_ntrC*_* (300 bp-fragment upstream of the start codon or the putative TSS, respectively) to *eGFP* were tested in Sm2B3001 grown in rich medium (TY) and N-limiting media, *i.e.,* MM and MM with 0.5 mM NH_4_^+^ as nitrogen source (left bar graph). Fluorescence values of three independent transconjugants were normalized to the culture OD_600_. Bacteria expressing *ntrC^FLAG^* from P*_dusB_* or P*_ntrC*_* were grown in the same media to logarithmic and stationary phase, and lysates were subjected to Western blot analysis to detect the FLAG-tagged protein (right panel). All gel lanes were loaded with equal protein amounts (OD_600_ equivalent to 0.05). (B) NtrC binds P*_dusB_*. Gel shift assays with radiolabeled P*_dusB_* (300 bp) incubated with increasing concentrations of purified NtrC as indicated on top of the panel. (C) RT-qPCR of NfeR1 and *ntrC* in free-living and nodule bacteria. Total RNA was extracted from bacteria grown in N-excess (10 mM NH_4_) or N-limiting (0.5 mM NH_4_) media, and from mature nodules harvested 28 days after plants inoculation. Relative Quantification (RQ) values were normalized to *SMc01852* as a constitutive control. Values plotted in the bar graphs are means and SD of two independent experiments.

**Table S1.** Bacterial strains and plasmids.

**Table S2.** Oligonucleotides.
