# Supplementary material for: A double-negative feedback loop between NtrBC and a small RNA rewires nitrogen metabolism in legume symbionts
Source: mBio. 2023 Oct 18;14(6):e02003-23. doi: 10.1128/mbio.02003-23 (PMC10746234; doi:10.1128/mbio.02003-23)
Supplement: Table S1 — Bacterial strains and plasmids. [file mbio.02003-23-s0006.docx]

| **Strain** | **Relevant characteristics** | **Reference/Source** |
| --- | --- | --- |
| ***S. meliloti*** |  |  |
| Sm2011 | SU47 derivative; Sm^r^ | [1] |
| Sm2B3001 | Sm2011 *expR^+^* derivative; Nal^r^, Sm^r^ | [2] |
| Sm2019 | Sm2B3001 derivative; markerless Δ*expR,*  ∆*sinRI*; Nal^r^, Sm^r^ | [3] |
| Sm2020 | Sm2019 ∆*abcR1*/*abcR2*/*nfeR1* derivative | [4] |
| SmΔ*lsrB* | Sm2011 ∆*lsrB* derivative; Sm^r^ | [4] |
| SmΔ*ntrC* | Sm2B3001 Δ*ntrC derivative*; Sm^r^ | This work |
| SmΔ*ntrB* | Sm2B3001 Δ*ntrB derivative*; Sm^r^ | This work |
| SmΔ*nfeR1* | Sm2B3001 Δ*nfeR1 derivative*; Sm^r^ | [5] |
| Sm2011-*mCherry* | 2011 labeled with *mCherry* by a single chromosomal integration of pKOSm | This work |
| Sm2011Δ*nfeR1-mCherry* | 2011 Δ*nfeR1* labeled with *mCherry* by a single chromosomal integration of pKOSm | This work |
| Sm2011-*egfp* | 2011 labeled with *egfp* by a single chromosomal integration of pKOSe | This work |
| Sm2011Δ*nfeR1-egfp* | 2011 Δ*nfeR1* labeled with *egfp* by a single chromosomal integration of pKOSe | This work |
| ***E. coli*** |  |  |
| DH5α | F^–^*endA1glnV44thi*  *1recA1relA1gyrA96deoRnupGpurB20*  φ80d*lacZ*ΔM15 Δ(*lacZYA-argF*)U169,  hsdR17(*r_K_*^–^*m_K_*^+^), λ^–^ | [6] |
| S17-1 | *recA pro hsdR RP4-2-Tc::Mu-Km::Tn7* | [7] |
| BL21(DE3) | *E. coli str. B F– ompT gal dcmlonhsdS_B_(r_B_^–^m_B_^–^) λ(DE3 [lacI lacUV5-T7p07 ind1 sam7 nin5])*  *[malB^+^]_K-12_(λ^S^)* | Novagen |
| **Plasmids** | **Relevant characteristics** | **Reference/Source** |
| pK18*mobsacB* | Suicide plasmid in *S. meliloti*, *sacB*, *oriV*, Km^r^ | [8] |
| pK18Δ*lsrB* | Suicide plasmid for *lsrB* deletion; Km^r^ | [4] |
| pK18Δ*ntrC* | Suicide plasmid for *ntrC* deletion; Km^r^ | This work |
| pK18Δ*ntrB* | Suicide plasmid for *ntrB* deletion; Km^r^ | This work |
| pET16b | Bacterial vector for inducible expression of N-terminally 10xHis-tagged proteins with a Factor Xa site | Novagen |
| p16*lsrB* | pET16b derivate carrying N-terminally 10xHis-tagged LsrB | [4] |
| pET29a | Bacterial vector for expression of N-terminally S-tagged proteins with a thrombin site | Novagen |
| p29*ntrC* | pET29a derivate carrying NtrC | This work |
| pSRKKm | pBBR1MCS-2 derivative with a P*_lac_* promoter,  *lacIq, lacZa*^+^, Km^r^ | [9] |
| pSRKKm-NtrC | pSRKKm derivate carrying the *ntrC* CDS and IPTG-induced expression of protein; Km^r^ | This work |
| pSKiNfeR1 | pSRKKm carrying the NfeR1 coding sequence fused to *sinR*-P*_sinI_* | [5] |
| pSKiNfeR1abc | pSRKKm derivatives expressing NfeR1 mutants in loops a, b and c | [5] |
| pBB-*eGFP* | pBBR1MCS-2 derivative for generation of promoter eGFP fusions; Km^r^ | [5] |
| pBBNfeR1-40*::eGFP* | pBBR1MCS-2 derivative expressing a transcriptional fusion of a truncated *nfeR1* promoter (54-bp) to *eGFP*; Km^r^ | [5] |
| pBBNfeR1-100*::eGFP* | pBBR1MCS-2 derivative expressing a transcriptional fusion of a truncated *nfeR1* promoter (114 bp) to *eGFP*; Km^r^ | [5] |
| pBBNfeR1-213:*:eGFP* | pBBR1MCS-2 derivative expressing a transcriptional fusion of a full-length *nfeR1* promoter (227-bp) to *eGFP p*; Km^r^ | This work |
| pBBNfeR1-100*::*eGFP* | pBBR1MCS-2 derivative expressing a transcriptional fusion of a *nfeR1* promoter (114-bp) including mutations to *eGFP*; Km^r^ | This work |
| pBBNfeR1-213*::*eGFP* | pBBR1MCS-2 derivative expressing a transcriptional fusion of a *nfeR1* promoter (227-bp) including mutations to *eGFP*; Km^r^ | This work |
| pBBP*_glnII_*:*:eGFP* | pBBR1MCS-2 derivative expressing a transcriptional fusion of a full-length *glnII* promoter (400-bp) to *egfp*; Km^r^ | This work |
| pBBP*_dusB_*::*eGFP* | pBBR1MCS-2 derivative expressing a transcriptional fusion of a *dusB* promoter (243-bp) to *egfp*; Km^r^ | This work |
| pBBP*_ntrC_*::*eGFP* | pBBR1MCS-2 derivative expressing a transcriptional fusion of a predicted *ntrC* promoter (300-bp) to *egfp*; Km^r^ | This work |
| pABCa | *oriVSm* (repABCpMlb); *oriVEc* (*E. coli* *oriVp15A*); AR (P*_min2_*- *aacC1*); synTer-MCS (synTer1); Gm^r^ | [10] |
| pABCaNfeR1-40 | pABCa::*GFP* derivate expressing a transcriptional fusion of a truncated *nfeR1* promoter (54-bp) to *egfp*; Gm^r^ | This work |
| pABCaNfeR1-100 | pABCa::*GFP* derivate expressing a transcriptional fusion of a truncated *nfeR1* promoter (114 bp) to *egfp*; Gm^r^ | This work |
| pABCaNfeR1-213 | pABCa::*GFP* derivate expressing a transcriptional fusion of a full-length *nfeR1* promoter (227-bp) to *egfp*; Gm^r^ | This work |
| pABCaNfeR1-213* | pABCa::*GFP* derivate expressing a transcriptional fusion of a full-length *nfeR1* promoter (227-bp) including mutations to *egfp*; Gm^r^ | This work |
| pR-eGFP | Vector for generation of target mRNA-*egfp* translational fusions; Ap^r^, Tc^r^ | [11] |
| pR*ntrB::eGFP* | *ntrB::eGFP* translational fusion (-143/+48 relative to *ntrB* AUG); Ap^r^, Tc^r^ | This work |
| pR_FLAG | Vector carrying 3xFLAG for C-terminal protein tagging; Km^r^ | This work |
| pR*ntrBntrC*^FLAG^ | pR_FLAG expressing *ntrC*^FLAG^; Km^r^ | This work |
| pR*dusBntrBntrC*^FLAG^ | pR_FLAG expressing *ntrC*^FLAG^; Km^r^ | This work |
| pKOSm | pK18mobII with P_T5_:*mCherry* cassette fused to *recG* | [3] |
| pKOSe | pK18mobII with P_T5_:*egfp* cassette fused to *recG* | [3] |

**Table S1 References**

1. Casse F, Boucher C, Julliot JS, *et al* (1979) Identification and Characterization of Large Plasmids in *Rhizobium meliloti* using Agarose Gel Electrophoresis. J Gen Microbiol 113:229–242. https://doi.org/10.1099/00221287-113-2-229

2. Bahlawane C, McIntosh M, Krol E, Becker A (2008) *Sinorhizobium meliloti* regulator MucR couples exopolysaccharide synthesis and motility. Mol Plant Microbe Interact 21:1498–1509. https://doi.org/10.1094/MPMI-21-11-1498

3. Robledo M, Frage B, Wright PR, Becker A (2015) A stress-induced small RNA modulates alpha-rhizobial cell cycle progression. PLoS Genet 11:e1005153. https://doi.org/10.1371/journal.pgen.1005153

4. García-Tomsig NI, Robledo M, Dicenzo GC, *et al* (2022) Pervasive RNA Regulation of Metabolism Enhances the Root Colonization Ability of Nitrogen-Fixing Symbiotic a-Rhizobia. mBio 13(1):e0357621. https://doi.org/doi: 10.1128/mbio.03576-21

5. Robledo M, Peregrina A, Millán V, *et al* (2017) A conserved α‐proteobacterial small RNA contributes to osmoadaptation and symbiotic efficiency of rhizobia on legume roots. Environ Microbiol 19:2661–2680. https://doi.org/doi:10.1111/1462-2920.13757

6. Grant SGN, Jessee J, Bloom FR, Hanahan D (1990) Differential plasmid rescue from transgenic mouse DNAs into *Escherichia coli* methylation-restriction mutants. Proc Natl Acad Sci 87:4645–4649. https://doi.org/10.1073/PNAS.87.12.4645

7. Simon R, Priefer U, Pühler A (1983) A broad host range mobilization system for in vivo genetic engineering: transposon mutagenesis in gram negative bacteria. Nat Biotech 1:784–791

8. Schäfer A, Tauch A, Jäger W, et al (1994) Small mobilizable multi-purpose cloning vectors derived from the *Escherichia coli* plasmids pK18 and pK19: selection of defined deletions in the chromosome of *Corynebacterium glutamicum*. Gene 145:69–73

9. Khan SR, Gaines J, Roop RM, Farrand SK (2008) Broad-host-range expression vectors with tightly regulated promoters and their use to examine the influence of TraR and TraM expression on Ti plasmid quorum sensing. Appl Environ Microbiol 74:5053–5062. https://doi.org/10.1128/AEM.01098-08

10. Döhlemann J, Wagner M, Happel C, *et al* (2017) A Family of Single Copy repABC-Type Shuttle Vectors Stably Maintained in the Alpha-Proteobacterium *Sinorhizobium meliloti*. ACS Synth Biol 6:968–984. https://doi.org/ 10.1021/acssynbio.6b00320

11. Torres-Quesada O, Millán V, Nisa-Martínez R, *et al* (2013) Independent activity of the homologous small regulatory RNAs AbcR1 and AbcR2 in the legume symbiont *Sinorhizobium meliloti.* PLoS One 8:1080–1091. https://doi.org/10.1371/journal.pone.0068147
