# Supplementary material for: A double-negative feedback loop between NtrBC and a small RNA rewires nitrogen metabolism in legume symbionts
Source: mBio. 2023 Oct 18;14(6):e02003-23. doi: 10.1128/mbio.02003-23 (PMC10746234; doi:10.1128/mbio.02003-23)
Supplement: Table S2 — Oligonucleotides. [file mbio.02003-23-s0007.docx]

| **Oligonucleotides** | **5’-sequence-3’** | **Use** |
| --- | --- | --- |
| **PbNfeR1** | TGCTTGATCTGATTGGCAACCGGGA | Northern blot probing |
| **Pb5S** | TACTCTCCCGCGTCTTAAGACGAA |  |
| **BamHIntrBFw** | ATTAGGATTCCGGTCCATGGCGGACAAG | Construction and PCR verification of SmΔ*ntrC* mutant |
| **ATGXbaIRv** | CGAATCTAGACGCCCGTCATCCATTGGT |  |
| **XbaITGAFw** | GTTGTCTAGAAGCGCTTGACTGAGATGC |  |
| **ntrYHindIIIRv** | GCCGAAGCTTATTGCGCTCCAGGTCGTT |  |
| **ntrCoutFw** | CGGCCGATGAAGAAACTC |  |
| **ntrCoutRv** | TGTCATCAGCTCGACGAAG |  |
| **BamHIntrBupFw** | CGTGGGATCCTGAAGAATTCCGGCATCG | Construction and PCR verification of SmΔ*ntrB* mutant |
| **ntrBupXbaIRv** | CTACTCTAGACTTCTCGGTCATGCCGCA |  |
| **XbaIntrBdownFw** | CTTGTCTAGACCGGCATCCAAGGGGCTG |  |
| **ntrBdownHindIIIRv** | CTTGAAGCTTGCGCATCCATCGGCATGT |  |
| **ntrBoutFw** | TCGTGACCGAGATGGTG |  |
| **ntrBoutRv** | TGCGCCCGTCATCCATT |  |
| **P14C2EcoRIFw** | CGTAGAATTCCGGTTGCCAATCGCCT | P*_nfeR1-213_* amplification |
| **P14C2XbaIRv** | TGAGTCTAGAGCTGCCCGATCGATGA |  |
| **EcoRIPc14mutFw** | AATTCATTCTGTGATCATTCGGCGCCTGAGCCAacgATCACTacgATAGGTGCCATTCGCGGCAGCCCCTGGTAAAATCCGGGGGTTCGGCCTATATTCCAATCATCGATCGGGCAGCC | Generation of P*_nfeR1-100_** by annealing |
| **XhoIPc14mutRv** | TCGAGGCTGCCCGATCGATGATTGGAATATAGGCCGAACCCCCGGATTTTACCAGGGGCTGCCGCGAATGGCACCTATcgtAGTGATcgtTGGCTCAGGCGCCGAATGATCACAGAATG |  |
| **NtrCbsFw** | CGATCCACTTTTCCATTATTTCGTTATTCTGTGAT | Generation of P*_nfeR1-213_** |
| **NtrCbsRv** | AATAATGGAAAAGTGGATCG |  |
| **XbaImTSSglnIIR** | GAAGTCTAGATGAGCAAATCCTGCCGG | P*_glnII_* amplification |
| **HindIIIPglnII-400F** | GTTCAAGCTTATTGATGCAACGGCCGC |  |
| **HindIIIPdusB** | TATCAAGCTTTATTGCTTCGGTCCCTCG | P*_dusB_* amplification |
| **PdusBXbaIRv** | GGCCTCTAGAGAAAATCTTGCACTATT |  |
| **mTSSntrC300up** | CGTCAAGCTTTTGCTGACGACGGCCTAT | P*_ntrC_* amplification |
| **PntrCXbaIRv** | GCCGTCTAGATTTCTTCATCGGCCGCCA |  |
| **avrIISRFw** | CAATCCTAGGCACCGCGGGGAAGTACGCCA | Construction of pABCa::*GFP* transcriptional fusions |
| **avrIIGFPRv** | CGGCCCTAGGTTAGCAGCCGGATCCTTTGTATAG |  |
| **BtnPC14Fw** | BiotinCGGTTGCCAATCGCCTTTATGACGCC | DNA-chromatography pull down assay and construction of P*_nfeR1Δ_* probe |
| **PC14FusFw** | TTGCCCATTATTTCGGCGCCCCTGGTAAAATCCGGG |  |
| **PC14FusRv** | GCCGAAATAATGGGCAAGATCGTTATACAAAATGCG |  |
| **P14C2Rv** | TCTAGAGCTGCCCGATCGATGATTGG |  |
| **ntrB_Fw** | ACCAGGATCCTATCTCGATCGTTTCGC | Amplification of the *ntrB* 5’-region fused to *eGFP* |
| **ntrB_Rv** | TAATGCTAGCGGAAAGATCGTTGGCACC |  |
| **NtrC_Fw_NdeI** | ATATCATATGACGGGCGCAACGATCCT | Amplification of the NtrC CDS |
| **NtrC_Rv_HindIII** | ATAAGCTTATCAAGGCTACGCGAGCTGC |  |
| **ntrB957F** | CCCGTTCATCACCACCAAGA | qRT-PCR of *ntrB, ntrC, glnII, lsrB* and *SMc01852* |
| **ntrB1074R** | GAAGGTCGTGCGGCTATGCT |  |
| **ntrC172F** | CCGGATGAAAACGCCTTCG |  |
| **ntrC290R** | CCCTTCTCCGAAGCCTTGATG |  |
| **glnII500F** | AGGCATCAACGCCGAAGTG |  |
| **glnII614R** | GTTAGGCGCAGCAGAAGGTAG |  |
| **lsrB517F** | GCGCCGTCCTACATCAACAA |  |
| **lsrB631R** | GCCAGTTCACGTCGAGCAGA |  |
| **Smc01852F** | TCACCAACACTGCCGACTGC |  |
| **Smc01852R** | TCGTGTGCAGGATGCTGATG |  |
| **NheISphIFlagF** | CTAGCGCATGCGATTACAAGGATCACGATGGTGATTACAAGGATCACGATATCGATTACAAGGATGACGATGACAAGTGAG | Construction of pR_FLAG |
| **EcoRIFlagR** | AATTCTCACTTGTCATCGTCATCCTTGTAATCGATATCGTGATCCTTGTAATCACCATCGTGATCCTTGTAATCGCATGCG |  |
| **ntrBNheI** | GACCGCTAGCTCCATTGGTTCCTTTTGTC | Construction of *ntrB* fused to 3xFLAG |
| **ntrCNheI** | GATTGCTAGCAGCGCTACGCGAGCTGCG | Construction of *ntrC* fused to 3xFLAG |
| **PCR1** | CGGGCCTCTTCGCTATT | Sequencing |
| **PCR2** | TTAGCTCACTCATTAGG |  |
| **SR_Fw** | CTGATCGGCATCAGCGTCAC |  |
| **GFP_Rv** | GTTGGCCATGGAACAGGTAG |  |
| **pABC_Fw** | CTGTTGTTTGTCGGTGAACG |  |
| **pABC_Rv** | GCCAGTTACCTCGGTTCAAA |  |
| **Egfp-139_rev** | GATGAACTTCAGGGTCAGCTTG |  |
| **SP6** | GTATTCTATAGTGTCACCTAAATAGC |  |
| **T7** | TAATACGACTCACTATAGGGCGA |  |
